# An annotated checklist of the scale insects of Iran (Hemiptera, Sternorrhyncha, Coccoidea) with new records and distribution data

**DOI:** 10.3897/zookeys.334.5818

**Published:** 2013-09-23

**Authors:** Masumeh Moghaddam

**Affiliations:** 1 Insect Taxonomy Research Department, Iranian Research Institute of Plant Protection, Tehran 19395, P.O. Box 1454, Tehran-Iran

**Keywords:** Hemiptera, Sternorrhyncha, coccoidea, annotated checklist, host plants, Iran, new records, locality data

## Abstract

A list of scale insects (Hemiptera: Sternorrhyncha: Coccoidea) of Iran is present based mainly on the literature records since 1902. In total, 13 families and 275 species have been recorded and these are listed along with their locality data and host plants. The families are as follows: Asterolecaniidae, Cerococcidae, Coccidae, Diaspididae, Eriococcidae, Kermesidae, Margarodidae, Monophlebidae, Ortheziidae, Phoenicococcidae, Pseudococcidae, Putoidae and Rhizoecidae. The following ten species are recorded for the first time from Iran: *Diaspidiotus lenticularis* (Lindinger), *Diaspidiotus wuenni* (Lindinger), *Fiorinia proboscidaria* Green, *Koroneaspis lonicerae* Borchsenius, *Eriococcus cingulatus* Kiritchenko, *Eriococcus pamiricus* (Bazarov), *Eriococcus reynei* Schmutterer, *Eriococcus sanguinairensis* Goux and *Eriococcus saxidesertus* (Borchsenius) and *Porphyrophora victoriae* Jashenko.

## Introduction

Scale insects (superfamily Coccoidea) are sap-sucking hemipterous insects with an estimated 8000 species within 49 families, of which 16 are only known from fossils (Ben-Dov et al. 2013). They are closely related to the other Sternorrhyncha superfamilies: aphids (Aphidoidea), whiteflies (Aleyrodoidea) and jumping plant lice (Psylloidea) (Gullan and Cook 2007). The taxonomy of the Coccoidea is mainly based on the microscopic cuticular features of the adult female, which is paedomorphic, maturing to a wingless juvenile form with functional mouthparts, whereas the adult male (when present) goes through “prepupal” and “pupal” stages and turns into an alate form with non-functional mouthparts (Kondo et al. 2008). The size of adult females varies from 1 mm in diameter in some diaspidids to 7−9 mm in several monophlebids. Some species are highly cryptic, living well hidden on their host plants, and are very hard to detect. This is particularly true of the immature stages, and these are frequently imported, causing major phytosanitary concerns. Many species of scale insects are economically important pests in agriculture, horticulture and forestry (Ben-Dov 2011–2012). However, only 13 families of scale insects have so far been recorded from Iran.

Although the scale insects of Iran have been relatively well studied, there is still a strong need for further investigations, including extensive collections of these families in Iran. Historically, the first scale insect recorded from Iran was *Asterolecanium bornmuelleri* (Asterolecaniidae), associated with its host plant *Quercus persica* (Rüebsaamen 1902). Lindinger (1905) wrote briefly on Iranian scale insects, describing *Salicicola kermanensis* on poplars. Subsequently Lindinger (1911) described *Parlatoria ephedrae* on *Ephedra nebrodenis* and recorded *Aonidiella orientalis* Newstead, on *Ficus* sp., and later he also reported *Diaspis syriaca* on *Pistacia* sp. (Lindinger 1931). Earlier, Green (1919) had recorded *Trabutina serpentina* on *Tamarix* sp., and later Archangelskaya (1937) recorded the following 12 species from Iran: *Orthezia urticae* (Linnaeus), *Palaeolecanium bituberculatum* (Signoret), *Eulecanium tiliae* (Linnaeus), *Sphaerolecanium prunastri* (Boyer de Fonscolombe), *Rhizopulvinaria artemisiae* (Signoret), *Pulvinaria vitis* (Linnaeus), *Anapulvinaria pistaciae* (Bodenheimer), *Parlatoria blanchardi* Targioni Tozzetii, *Parlatoria oleae* Colvée, *Lepidosaphes beckii* (Newman), *Lepidosaphes pistaciae* Archangelskaya and *Chlidaspis asiatica* (Archangelskaya). Afchar (1937) listed 31 scale insects from Iran. Bodenheimer (1944) was the first coccidologist to make a solo expedition to Iran, and he assembled a sizeable collection of Iranian coccoids, recording 34 species, of which two were new: *Pulvinaria gossypii* (Bodenheimer), and *Epidiaspis salicis* (Bodenheimer). Borchsenius (1952) added two further new species, *Targionia anabasidis* (Borchsenius) and *Dynaspidiotus amygdalicola* Borchsenius. Balachowsky between the years 1937 and 1967 made the greatest contribution to the taxonomy of diaspidids in Iran, and jointly worked with other Iranian entomologists to significantly improve the understanding of the scale insect fauna of the country. Kaussari was the first Iranian coccidologist to extensively revise the diaspidid fauna of Iran, between 1946 and 1970. Later contributions are those of Farahbakhsh (1961), Asadeh and Mosaddegh (1991), Vahedi from 2001 through 2012, Vahedi and Gholami Mahfar (2010), Vahedi and Hodgson (2002, 2002a, 2007), Vahedi and Hodjat (1996), Torabi et al. (2010), Moghaddam (1999 to 2013), Moghaddam and Alikhani (2009, 2010), Moghaddam and Azadbakht (2006), Moghaddam and Bagheri (2011), Moghaddam and Tavakoli (2010), Moghaddam et al. (2004), Takagi and Moghaddam (2005) and Williams and Moghaddam (1999, 2007).

Additional information has been gleaned from the various catalogues by Borchsenius (1966), Ben-Dov (1993, 1994), Kozár (1998), Kozár and Walter (1985), Miller and Gimpel (2000) and the check-lists of Iranian Coccoidea by Afchar (1937), Kozár et al. (1996), Seghatoleslami (1977) and Moghaddam (2004, 2009).

This present checklist is intended to facilitate access to the most recent data on Iranian Coccoidea for taxonomists and to update the recorded species from Iran. Only records in which Iran is specifically mentioned are cited.

The list contains all species of Coccoidea recorded up to March, 2013 and includes 275 species in 113 genera and 13 families: Asterolecaniidae (seven species.), Cerococcidae (one species), Coccidae (30 species), Diaspididae (151 species), Eriococcidae (14 species), Kermesidae (two species), Margarodidae (seven species), Monophlebidae (five species), Ortheziidae (one species), Phoenicococcidae (one species), Pseudococcidae (54 species), Putoidae (one species) and Rhizoecidae (one species). New records from Iran are marked with asterisks include *Diaspidiotus lenticularis* (Lindinger), *Diaspidiotus wuenni* (Lindinger), *Fiorinia proboscidaria* Green, *Koroneaspis lonicerae* Borchsenius, *Eriococcus cingulatus* Kiritchenko, *Eriococcus pamiricus* (Bazarov), *Eriococcus reynei* Schmutterer, *Eriococcus sanguinairensis* Goux, *Eriococcus saxidesertus* (Borchsenius) and *Porphyrophora victoriae* Jashenko.

The following 32 species are currently only known from Iran: *Asterolecanium bornmuelleri* Rübsaamen, *Acanthomytilus kurdicus* (Bodenheimer), *Aspidaspis dentilobus* Kaussari & Balachowsky, *Chorizococcus viticola* Kaydan & Kozár, *Chortinaspis salavatiani* Balachowsky & Kaussari, *Coccidohystrix burumandi* Moghaddam, *Contigaspis davatchii* Kaussari, *Contigaspis sarkissiani* (Kaussari & Balachowsky), *Diaspidiotus baiati* (Kaussari), *Diaspidiotus iranicus* Kaussari & Balachowsky, *Diaspidiotus platychaetae* Takagi & Moghaddam, *Diaspis carmanica* Davatchi & Balachowsky, *Dynaspidiotus amygdalicola* (Borchsenius), *Dynaspidiotus medicus* Kaussari, *Eriococcus abaii* (Danzig), *Exallomochlus balouchestanensis* Moghaddam, *Melanaspis louristanus* Balachowsky & Kaussari, *Parlagena mckenziei* Balachowsky, *Parlagena remaudierei* Kaussari, *Peliococcus ilamicus* Moghaddam, *Phenacoccus betae* Moghaddam, *Phenacoccus iranica* Moghaddam, *Phenacoccus karkasicus* Moghaddam, *Phenacoccus salviacus* Moghaddam, *Polystomophora arakensis* Moghaddam, *Porphyrophora chelodonta* Vahedi, *Pseudotargionia orientalis* Balachowsky & Kaussari, *Rhodania aeluropi* Williams & Moghaddam, *Rungaspis avicenniae* Takagi & Moghaddam, *Spilococcus mirzayansi* (Moghaddam), *Targionia balachowskyi* (Kaussari), *Torosapis farsianus* (Balachowsky & Kaussari), These possibly endemic species are marked with black spots.

## Notes on the checklist

**Figure 1.**
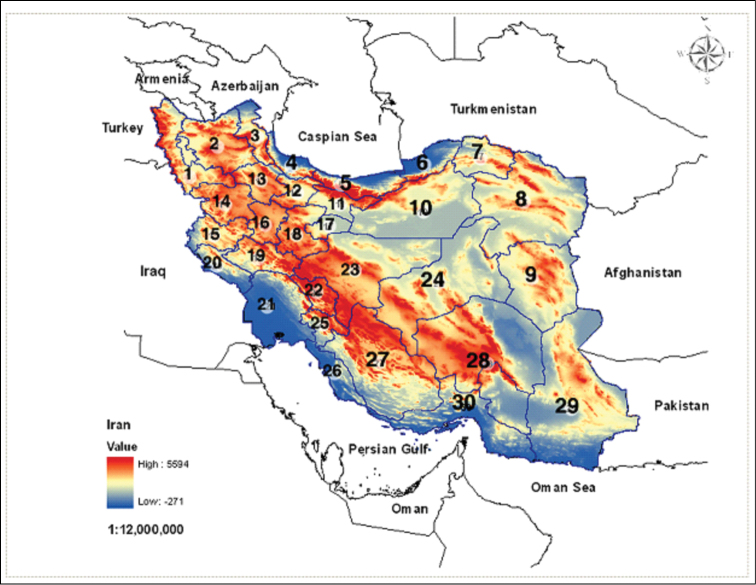
Outline map of Iranian provinces. **1** Azarbaijan - e Garbi **2** Azarbaijan -e Shargi **3** Ardabil **4** Gilan **5** Mazandaran **6** Golestan **7** Khorosan -e Shomali **8** Khorasan -e Razavi **9** Khorasan -e Jonoubi **10** Semnan **11** Tehran **12** Ghazvin **13** Zanjan **14** Kordestan **15** Kermanshah **16** Hamadan **17** Qom **18** Markazi **19** Lorestan **20** Ilam **21** Khouzestan **22** Chaharmahal-Bakhtiari **23** Esfahan **24** Yazd **25** Kohgilouyeh & Boyerahmad **26** Bushehr **27** Fars **28** Kerman **29** Sistan & Belouchestan **30** Hormozgan

The families are alphabetically ordered and are diagnosed based on the most recent classification of Coccoidea by Ben-Dov et al. (2013), except for Rhizoecidae that follows Hodgson (2012). The genera and species are listed under their families and ordered alphabetically, together with related references, host plants and collecting sites in Iran (Fig. 1). Further information is provided if there are either new host plant or new species records. Full reference citations can be found on ScaleNet (Ben-Dov et al. 2013).

Slide-mounted specimens of most of the species are deposited in the Coccoidea collection, Insect Taxonomy Research Department, Iranian Research Institute of Plant Protection (IRIPP), Tehran, Iran. Collections are also housed in the Natural History Museum, London, UK (BMNH); Muséum National d’Histoire Naturelle, Paris, France (MNHN); United States National Entomological Collection, U.S. National Museum of Natural History, Washington, D.C., USA (USNM); Department of Entomology, The Volcani Center, Bet Dagan, Israel (ICVI); Zoological Museum, Academy of Science, St. Petersburg, Russia (ZIRAS), Plant Protection Department, Faculty of Agriculture, Yuzuncu Yil University, Van, Turkey and Zoologisches Institut und Zoologisches Museum, Hamburg, Germany (ZMUH).

## Family ASTEROLECANIIDAE

### Asterodiaspis bella

(Russell)

Asterolecanium bellum Russell, 1941: 50.

#### Iran localities.

Unknown.

#### Host plants.

Fagaceae: *Quercus* sp.

#### References.

Ben-Dov et al. (2013), Kozár et al. (1996).

### Asterodiaspis mina

(Russell)

Asterolecanium minus Russell, 1941: 132.

#### Iran localities.

Fars, Golestan.

#### Host plants.

Fagaceae: *Quercus infectoria*.

#### References.

Ben-Dov et al. (2013), Bodenheimer (1943, 1944), Farahbakhsh (1961), Kozár et al. (1996), Moghaddam (2009), Moghaddam and Tavakoli (2010).

### Asterodiaspis quercicola

(Bouché)

Lecanium quercicola Bouché, 1851: 112. *Asterolecanium quercicola* Signoret, 1870.

#### Iran localities.

Chaharmahal-Bakhtiari, Fars, Ilam, Kermanshah, Khouzestan, Kohgilouyeh & Boyerahmad, Kordestan, Lorestan.

#### Host plants.

Fagaceae: *Quercus* sp.

#### References.

Ben-Dov et al. (2013), Bodenheimer (1943), Farahbakhsh (1961), Kaussari (1957), Moghaddam (2009, 2010), Moghaddam and Tavakoli (2010).

### ● Asterolecanium bornmuelleri

Rübsaamen

Asterolecanium bornmuelleri Rübsaamen, 1902: 316.

#### Iran localities.

Fars.

#### Host plants.

Fagaceae: *Quercus persica*.

#### References.

Ben-Dov et al. (2013), Bodenheimer (1944), Fernald (1903), Kozár (1998), Kozár and Drozdják (1998), Lindinger (1912), Rübsaamen (1902), Russell (1941).

### Bambusaspis bambusae

(Boisduval)

Asterolecanium bambusae Boisduval, 1869: 261.

#### Iran localities.

Gilan.

#### Host plants.

Poaceae: *Bambusa* sp.

#### References.

Farahbakhsh (1961).

### Palmaspis phoenicis

(Ramachandra Rao)

Asterolecanium phoenicis Ramachandra Rao, 1922: 11.

#### Iran localities.

Bushehr, Esfahan, Fars, Khorasan -e Jonoubi, Sistan & Balouchestan, Yazd.

#### Host plants.

Arecaceae: *Phoenix dactylifera*.

#### References.

Ben-Dov et al. (2013), Bodenheimer (1944), Borchsenius (1960), Farahbakhsh (1961), Kaussari (1957), Kozár (1998), Kozár et al. (1996), Moghaddam (2009).

### Russellaspis pustulans

(Cockerell)

Asterodiaspis pustulans Cockerell, 1892: 142.

#### Iran localities.

Unknown locality.

#### Host plants.

Unknown plant.

#### References.

Ben-Dov et al. (2013), Kozár (1998), Kozár et al. (1996), Stumpf and Lambdin (2006).

## Family CEROCOCCIDAE

### Cerococcus longipilosus

(Archangelskaya)

Cerveoccus longipilosus Archangelskaya, 1930a: 81.

#### Iran localities.

Ilam, Lorestan, Yazd.

#### Host plants.

Asteraceae: *Lactuca orientalis*.

#### References.

Ben-Dov et al. (2013), Kaussari (1957), Kozár (1998), Kozár et al. (1996), Moghaddam (2009, 2010), Moghaddam and Tavakoli (2010).

## Family COCCIDAE

### Acantholecanium haloxyloni

(Hall)

Ctenochiton haloxyloni Hall, 1926: 17.

#### Iran localities.

Azarbaijan -e Garbi, Esfahan, Semnan, Yazd.

#### Host plants.

Amaranthaceae: *Haloxylon* sp., *Noaea mucronata*.

#### References.

Ben-Dov et al. (2013), Moghaddam (2009).

### Acanthopulvinaria orientalis

(Nazonov)

Pulvinaria orientalis Nasonov, 1908: 493.

#### Iran localities.

Esfahan, Hamadan, Golestan, Kerman, Khorasan -e Shomali, Sistan & Balouchestan.

#### Host plants.

Amaranthaceae: *Halocnemum strobilaceum*, *Haloxylon* sp., *Noaea mucronata*, *Salsola oppositifolia*; Asteraceae: *Achillea* sp., *Artemisia* sp.; Rosaceae: *Prunus lycioides*; Tamaricaceae: *Tamarix* sp.

#### References.

Ben-Dov et al. (2013), Kozár (1998), Kozár et al. (1996), Moghaddam (2009, 2010), Moghaddam and Tavakoli (2010).

#### Notes.

This is the first record for *Acanthopulvinaria orientalis* from the plant family Rosaceae.

### Anapulvinaria pistaciae

(Bodenheimer)

Pulvinaria pistaciae Bodenheimer, 1926: 189.

#### Iran localities.

Hormozgan, Kerman, Khorasan -e Jonoubi, Sistan & Balouchestan, Yazd.

#### Host plants.

Anacardiaceae: *Pistacia khinjuk*, *Rhus coriaria*; Juglandaceae: *Juglans regia*; Tamaricaceae: *Tamarix* sp.

#### References.

Ben-Dov et al. (2013), Bodenheimer (1944), Farahbakhsh (1961), Kaussari (1957), Kozár (1998), Kozár et al. (1996), Moghaddam (2009).

#### Notes.

This is the first record of *Anapulvinaria pistaciae* from the plant family Juglandaceae.

### Bodenheimera rachelae

(Bodenheimer)

Lecanium (Eulecanium) racheli Bodenheimer, 1924: 68.

#### Iran localities.

Ilam, Kermanshah, Lorestan.

#### Host plants.

Lamiaceae: *Vitex* cf. *Pseudonegundo*.

#### References.

Ben-Dov (1969), Ben-Dov et al. (2013), Bodenheimer (1944), Kozár (1998), Kozár et al. (1996), Moghaddam (2009), Moghaddam and Tavakoli (2010).

### Ceroplastes floridensis

Comstock

Ceroplastes floridensis Comstock, 1881: 331.

#### Iran localities.

Ardabil, Fars, Gilan, Mazandaran.

#### Host plants.

Araliaceae: *Hedera pastuchowii*; Ebenaceae: *Diospyros kaki*; Moraceae: *Ficus benjamina*; Pinaceae: *Cedrus* sp.; Rosaceae: *Cydonia oblonga*; Rutaceae: *Citrus sinensis*; Taxodiaceae: *Metasequoia glyptostroboides*.

#### References.

Ben-Dov et al. (2013), Farahbakhsh (1961), Kaussari (1957), Kozár (1998), Kozár et al. (1996) and Moghaddam (2009, 2010).

#### Notes.

These are the first records of *Ceroplastes floridensis* from plant families Moraceae and Taxodiaceae.

### Ceroplastes rusci

(Linnaeus)

Coccus rusci Linnaeus, 1758: 456.

#### Iran localities.

Fars, Kohgilouyeh & Boyerahmad, Lorestan, Sistan & Balouchestan.

#### Host plants.

Moraceae: *Ficus carica*.

#### References.

Ben-Dov et al. (2013), Farahbakhsh (1961), Kaussari (1946a), Kozár et al. (1996), Moghaddam (2009) and Moghaddam and Tavakoli (2010).

### Ceroplastes sinensis

Del Guercio

Ceroplastes sinensis Del Guercio, 1900: 3.

#### Iran localities.

Gilan, Mazandaran.

#### Host plants.

Lythraceae: *Punica granatum*; Rosaceae: *Rosa* sp.

#### References.

Ben-Dov et al. (2013), Bodenheimer (1944), Farahbakhsh (1961), Kaussari (1946, 1957), Kozár (1998), Kozár et al. (1996), Moghaddam (2009, 2010).

#### Notes.

This is the first record of *Ceroplastes sinensis* from the plant family Lythraceae

### Coccus hesperidum

Linnaeus

Coccus hesperidum Linnaeus, 1758: 455.

#### Iran localities.

Elborz, Esfahan, Fars, Gilan, Golestan, Khouzestan, Markazi, Mazandaran, Sistan & Balouchestan, Tehran.

#### Host plants.

Apocynaceae: *Nerium oleander*; Aquifoliaceae: *Ilex* sp.; Asparagaceae: *Yucca baccata*; Fabaceae: *Alhagi camelorum*, *Cercis siliquastrum*, *Robinia pseudo-acacia*; Lauraceae: *Laurus nobilis*; Lycopodiaceae: *Lycopodium clavatum*; Lythraceae: *Punica granatum*; Moraceae: *Ficus benjamina*, *Ficus carica*, *Morus alba*; Myrsinaceae: *Cyclamen coum*; Nyctaginaceae: *Mirabilis jalapa*; Rosaceae: *Prunus armeniaca*; Rutaceae: *Citrus sinensis*, Ulmaceae: *Ulmus campestris*.

#### References.

Ben-Dov et al. (2013), Bodenheimer (1944), Farahbakhsh (1961), Kaussari (1946, 1957), Kozár et al. (1996), Moghaddam (2009, 2010) and Torabi et al. (2010).

#### Notes.

These are the first records of *Coccus hesperidum* from the plant families Aquifoliaceae, Asparagaceae, Lycopodiaceae, Moraceae and Rosaceae.

### Coccus pseudomagnoliarum

(Kuwana)

Lecanium (Eulecanium) pseudomagnoliarum Kuwana, 1914: 7.

#### Iran localities.

Golestan.

**Host plant.** Rutaceae: *Citrus* sp.

#### References.

Ben-Dov (1993), Ben-Dov et al. (2013), Farahbakhsh (1961), Kaussari (1946, 1957), Kozár (1998) and Kozár et al. (1996).

### Didesmococcus unifasciatus

(Archangelskaya)

Physokermes unifasciatus Archangelskaya, 1923: 265.

#### Iran localities.

Azarbaijan -e Sharghi, Kermanshah, Markazi.

#### Host plants.

Rosaceae: *Prunus amygdalus*, *Prunus persica*.

#### References.

Ben-Dov et al. (2013), Kozár et al. (1996), Moghaddam (2009) and Moghaddam and Tavakoli (2010).

### Eriopeltis festucae

(Boyer de Fonscolombe)

Coccus festucae Boyer de Fonscolombe, 1834: 216.

#### Iran localities.

Chaharmahal-Bakhtiari, Mazandaran, Zanjan.

#### Host plants.

Poaceae.

#### References.

Ben-Dov et al. (2013), Bodenheimer (1944), Kozár et al. (1996), Moghaddam (2009).

### Eulecanium ficiphilum

Borchsenius

Eulecanium ficiphilum Borchsenius, 1955: 293.

#### Iran localities.

Azarbaijan -e Garbi.

#### Host plants.

Unknown plant.

#### References.

Ben-Dov et al. (2013), Kozár (1998) and Kozár et al. (1996).

### Eulecanium rugulosum

(Archangelskaya)

Lecanium rugulosum Archangelskaya, 1937: 46.

#### Iran localities.

Fars, Kermanshah.

#### Host plants.

Anacardiaceae: *Pistacia* sp.; Betulaceae: *Corylus avellana*.

#### References.

Ben-Dov et al. (2013), Farahbakhsh (1961), Kaussari (1957), Kozár et al. (1996) and Moghaddam (2009).

#### Notes.

This is the first record of *Eulecanium rugulosum* from the plant family Betulaceae.

### Eulecanium tiliae

(Linnaeus)

Coccus tiliae Linnaeus, 1758: 456. *Eulecanium coryli* Cockerell, 1901 (nomen nudum).

#### Iran localities.

Esfahan. Kerman, Kermanshah, Sistan & Balouchestan, Tehran.

#### Host plants.

Anacardiaceae: *Pistacia khinjuk*; Rosaceae: *Cydonia oblonga*, *Malus domestica*, *Prunus caspica*, *Prunus reutri*.

#### References.

Ben-Dov et al. (2013), Bodenheimer (1944), Davoodi et al. (2002), Farahbakhsh (1961), Kaussari (1957), Kozár (1998), Kozár et al. (1996), Moghaddam (2009) and Moghaddam and Tavakoli (2010).

### Exaeretopus tritici

Williams

Exaeretopus tritici Williams, 1977: 281.

#### Iran localities.

Lorestan.

#### Host plants.

Poaceae: *Hordeum* sp.

#### References.

Ben-Dov et al. (2013), Moghaddam (2009), Moghaddam and Azadbakht (2006) and Moghaddam and Tavakoli (2010).

### Hadzibejliaspis stipae

(Hadzibejli)

Exaeretopus stipae Hadzibejli, 1960: 310.

#### Iran localities.

Kermanshah.

#### Host plants.

Poaceae.

#### References.

Ben-Dov et al. (2013) and Torabi et al. (2010).

### Palaeolecanium bituberculatum

(Signoret)

Lecanium bituberculatum Signoret, 1873a: 414. *Eulecanium bituberculatum* Fernald, 1903 (nomen nudum).

#### Iran localities.

Azarbaijan -e Garbi, Azarbaijan -e Sharghi, Esfahan, Kerman, Kermanshah.

#### Host plants.

Moraceae: *Morus alba*; Rosaceae: *Crataegus azarollus*, *Malus domestica*.

#### References.

Afchar (1937), Ben-Dov et al. (2013), Bodenheimer (1944), Farahbakhsh (1961), Kaussari (1957), Kozár (1998) and Moghaddam (2009).

#### Notes.

This is the first record of *Palaeolecanium bituberculatum* from the plant family Moraceae.

### Parthenolecanium corni

(Bouché)

Lecanium corni Bouché, 1844: 298. *Eulecanium corni* Fernald, 1903.

#### Iran localities.

Azarbaijan -e Garbi, Tehran.

#### Host plants.

Betulaceae: *Corylus avellana*; Oleaceae: *Fraxinus excelsior*; Solanaceae: *Solanum tuberosum*.

#### References.

Afchar (1937), Ben-Dov (1993), Ben-Dov et al. (2013), Kaussari (1957), Kozár (1998) and Moghaddam (2009).

#### Notes.

This is the first record of *Parthenolecanium corni* from the plant family Solanaceae.

### Parthenolecanium persicae

(Fabricius)

Chermes persicae Fabricius, 1776: 304. *Lecanium persicae* Bouché, 1844.

#### Iran localities.

Esfahan, Kermanshah, Tehran.

#### Host plants.

Moraceae: *Morus alba*.

#### References.

Ben-Dov et al. (2013), Bodenheimer (1944), Farahbakhsh (1961), Kaussari (1957), Kozár et al. (1996) and Moghaddam (2009).

### Pulvinaria aurantii

Cockerell

Pulvinaria aurantii Cockerell, 1896: 19. *Chloropulvinaria aurantii* Borchsenius, 1952.

#### Iran localities.

Gilan, Golestan, Mazandaran, Sistan & Balouchestan.

#### Host plants.

Moraceae: *Morus alba*; Myrtaceae: *Psidium guajava*; Rutaceae: *Citrus bigaradia*, *Citrus sinensis*.

#### References.

Ben-Dov et al. (2013), Bodenheimer (1944), Farahbakhsh (1961), Kaussari (1957), Kozár (1998), Kozár et al. (1996) and Moghaddam (2009, 2010).

#### Notes.

These are the first records of *Pulvinaria aurantii* from the plant families Moraceae and Myrtaceae.

### Pulvinaria floccifera

(Westwood)

Coccus flocciferus Westwood, 1870: 308. *Chloropulvinaria floccifera* Borchsenius, 1952.

#### Iran localities.

Mazandaran, Gilan.

#### Host plants.

Aquifoliaceae: *Ilex spinigera*; Rosaceae: *Mespilus germanica*.

#### References.

Afchar (1937), Ben-Dov et al. (2013), Bodenheimer (1944), Davatchi (1946), Farahbakhsh (1961), Kaussari (1957), Kozár (1998), Kozár et al. (1996) and Moghaddam (2009, 2010).

### ● Pulvinaria gossypii

(Bodenheimer)

Filippia gossypii Bodenheimer, 1944: 89.

#### Iran localities.

Sistan & Balouchestan.

#### Host plants.

Malvaceae: *Gossypium* sp.

#### References.

Ben-Dov et al. (2013), Bodenheimer (1944), Farahbakhsh (1961), Kaussari (1946, 1957), Kozár (1998) and Kozár et al. (1996).

### Pulvinaria vitis

(Linnaeus)

Coccus vitis Linnaeus, 1758: 455. *Pulvinaria betulae* Signoret, 1873

#### Iran localities.

Esfahan, Fars, Gilan, Ilam, Kermanshah, Khorasan -e Razavi, Mazandaran, Tehran, Yazd.

#### Host plants.

Betulaceae: *Alnus* sp.; Fabaceae: *Robinia* sp.; Moraceae: *Ficus carica*; Rosaceae: *Pyrus communis*; Salicaceae: *Salix* sp.; Vitaceae: *Vitis persica*.

#### References.

Afchar (1937), Ben-Dov et al. (2013), Bodenheimer (1944), Farahbakhsh (1961), Kaussari (1946, 1957), Kozár (1998), Kozár et al. (1996) and Moghaddam (2009, 2010).

#### Notes.

These are the first records of *Pulvinaria vitis* from the plant families Fabaceae and Moraceae.

### Rhizopulvinaria artemisiae

(Signoret)

Pulvinaria artemisiae Signoret, 1873: 31. *Rhizopulvinaria minima* Borchsenius, 1952. *Rhizopulvinaria virgulata* Borchsenius, 1952.

#### Iran localities.

Ardabil, Azarbaijan -e Sharghi, Chaharmahal-Bakhtiari, Esfahan, Fars, Golestan, Kerman, Kermanshah, Kohgilouyeh & Boyerahmad, Kordestan, Lorestan, Yazd and Zanjan.

#### Host plants.

Amaranthaceae: *Noaea mucrunata*; Asteraceae: *Achillea* sp., *Echinops ritro*, *Carthamus oxyacantha*; Fabaceae: *Astragalus* sp.; Rosaceae: *Prunus scoparia*.

#### References.

Ben-Dov et al. (2013), Bodenheimer (1944), Farahbakhsh (1961), Kozár (1998), Kozár et al. (1996) and Moghaddam (2001, 2009, 2010).

### Rhizopulvinaria turkestanica

(Archangelskaya)

Pulvinaria artemisiae turkestanica Archangelskaya, 1931: 81.

#### Iran localities.

Ardebil, Ilam, Kermanshah, Khorasan -e Shomali, Kordestan, Lorestan.

#### Host plants.

Amaranthaceae: *Noaea mucronata*; Asteraceae: *Carthamus oxyacantha*.

#### References.

Moghaddam and Tavakoli (2010).

### Rhodococcus turanicus

(Archangelskaya)

Lecanium coryli turanicum Archangelskaya, 1937: 47.

#### Iran localities.

Unknown.

#### Host plants.

Unknown plant.

#### References.

Ben-Dov et al. (2013), Kozár (1998) and Kozár et al. (1996).

### Saissetia coffeae

(Walker)

Lecanium coffeae Walker, 1852: 1079. *Saissetia hemisphaerica* Hall, 1922.

#### Iran localities.

Gilan.

#### Host plants.

Celastraceae: *Evonymus* sp.; Cycadaceae: *Cycas* sp.

#### References.

Ben-Dov et al. (2013), Farahbakhsh (1961), Kaussari (1957) and Kozár et al. (1996).

### Saissetia oleae

(Olivier)

Coccus oleae Olivier, 1791: 95.

#### Iran localities.

Gilan, Mazandaran, Tehran.

#### Host plants.

Apocynaceae: *Nerium oleander*; Oleaceae: *Olea europea*.

#### References.

Ben-Dov et al. (2013), Farahbakhsh (1961), Kaussari (1957), Ahmad (1975), Kozár (1998), Kozár et al. (1996) and Moghaddam (2009, 2010).

### Sphaerolecanium prunastri

(Boyer de Fonscolombe)

Coccus prunastri Boyer de Fonscolombe, 1834: 211. *Eulecanium prunastri* Fernald, 1903.

#### Iran localities.

Azarbaijan -e Sharghi, Esfahan, Tehran, Yazd.

#### Host plants.

Rosaceae: *Prunus amygdalus*, *Prunus reuteri*, *Prunus scoparia*, *Prunus* sp.

#### References.

Ben-Dov et al. (2013), Bodenheimer (1944), Kaussari (1957), Kozár (1998), Kozár et al. (1996) and Moghaddam (2009, 2010).

### Stotzia ephedrae

(Newstead)

Lichtensia ephedrae Newstead, 1901: 83. *Filippia ephedrae* Lindinger, 1912.

#### Iran localities.

Esfahan, Fars, Kerman, Yazd.

#### Host plants.

Ephedraceae: *Ephedra* sp.

#### References.

Ben-Dov et al. (2013), Bodenheimer (1944), Farahbakhsh (1961), Kaussari (1957), Kozár (1998), Kozár et al. (1996) and Moghaddam (2009).

## Family DIASPIDIDAE

### Acanthomytilus intermittens

(Hall)

Lepidosaphes intermittens Hall, 1924: 7.

#### Iran localities.

Sistan & Balouchestan.

#### Host plants.

Poaceae.

#### References.

Ben-Dov et al. (2013), Balachowsky (1954), Borchsenius (1966), Farahbakhsh (1961), Kaussari (1955), Kozár and Walter (1985) and Kozár et al. (1996).

### ● Acanthomytilus kurdicus

(Bodenheimer)

Mytilococcus kurdicus Bodenheimer, 1943: 5.

#### Iran localities.

Kohgilouyeh & Boyerahmad, Esfahan, Fars, Gilan, Hormozgan, Sistan & Balouchestan, Tehran.

#### Host plants.

Aceraceae: *Acer cinerascens*; Fabaceae: *Prosopis spicigera*.

#### References.

Ben-Dov et al. (2013), Bodenheimer (1944), Borchsenius (1966), Kaussari (1964), Kozár (1998), Kozár et al. (1996), Moghaddam (2004), Moghaddam and Tavakoli (2010) and Seghatoleslami (1977).

### Aonidiella aurantii

(Maskell)

Aspidiotus aurantii Maskell, 1879: 199.

#### Iran localities.

Gilan, Golestan, Mazandaran, Tehran.

#### Host plants.

Lythraceae: *Punica granatum*; Rosaceae: *Amygdalus persica*; Oleaceae: *Olea europaea*; Rutaceae: *Citrus sinensis*.

#### References.

Afchar (1937), Ben-Dov et al. (2013), Bodenheimer (1944), Farahbakhsh (1961), Kaussari (1946, 1955), Kaussari and Farahbakhsh (1968), Kozár et al. (1996), Moghaddam (2004, 2010) and Seghatoleslami (1977).

#### Notes.

This is the first record of *Aonidiella aurantii* from the plant family Lythraceae.

### Aonidiella citrina

(Coquillett)

Aspidiotus citrinus Coquillett, 1891: 29.

#### Iran localities.

Gilan, Golestan, Mazandaran.

#### Host plants.

Cornaceae: *Cornus* sp.; Rutaceae: *Citrus bigaradia*, *Citrus limetta*.

#### References.

Ben-Dov et al. (2013), CAB International (1997), Farahbakhsh (1961), Kaussari (1955), Kaussari and Farahbakhsh (1968), Kozár (1998), Kozár et al. (1996), Moghaddam (2004, 2010) and Seghatoleslami (1977).

#### Notes.

This is the first record of *Aonidiella citrina* from the plant family Cornaceae

### Aonidiella orientalis

(Newstead)

Aspidiotus orientalis Newstead, 1894: 26.

#### Iran localities.

Bushehr, Fars, Hormozgan, Khouzestan, Sistan & Balouchestan.

#### Host plants.

Anacardiaceae: *Mangifera indica*; Apocynaceae: *Calotropis procera*, *Nerium oleander*, *Periploca aphylla*; Arecaceae: *Cocos nucifera*, *Phoenix dactylifera*; Boraginaceae: *Cordia myxa*; Fabaceae: *Albizia julibrissin*, *Ceratonia siliqua*, *Dalbergia sissoo*, *Prosopis spicigera*, *Tamarindus indica*; Lythraceae: *Punica granatum*; Musaceae: *Musa sapientum*; Myrtaceae: *Callistemon lophanthus*, *Myrtus communis*, *Psidium guajava*, *Syzygium aromaticum*; Rhamnaceae: *Ziziphus spina-christi*; Rutaceae: *Citrus bigaradia*, *Citrus limon*, *Citrus limetta*, *Citrus sinensis*; Salicaceae: *Salix* sp.; Sapotaceae: *Manilkara zapota*; Tamaricaceae: *Tamarix indica*.

#### References.

Ben-Dov et al. (2013), Bodenheimer (1944, 1944a), Borchsenius (1966), Farahbakhsh (1961), Kaussari (1946a, 1955), Kaussari and Farahbakhsh (1968), Kozár (1998), Kozár et al. (1996), Lindinger (1909), Moghaddam (2004), Rosen and DeBach (1979) and Seghatoleslami (1977).

#### Notes.

This is the first record of *Aonidiella orientalis* from the plant family Tamaricaceae.

### ● Aspidaspis dentilobus

Kaussari & Balachowsky

Aspidaspis dentilobus Kaussari & Balachowsky, 1953a: 99.

#### Iran localities.

Esfahan, Fars, Kerman, Kohgilouyeh & Boyerahmad.

#### Host plants.

Polygonaceae: *Atraphaxis spinosa*.

#### References.

Ben-Dov et al. (2013), Borchsenius (1966), Farahbakhsh (1961), Kaussari (1964, 1955), Kaussari and Balachowsky (1953b), Kaussari and Farahbakhsh (1968), Kozár (1998), Kozár et al. (1996), Moghaddam (2004) and Seghatoleslami (1977).

### Aspidiotus destructor

Signoret

Aspidiotus destructor Signoret, 1869: 120.

#### Iran localities.

Khouzestan, Sistan & Balouchestan.

#### Host plants.

Arecaceae: *Nannorrhops ritchiana*; Musaceae: *Musa sapientum*.

#### References.

Ben-Dov et al. (2013), Farahbakhsh (1961), Kaussari (1955), Kaussari and Farahbakhsh (1968), Kozár (1998) and Moghaddam (2004).

### Aspidiotus nerii

Bouché

Aspidiotus nerii Bouché, 1833: 52.

#### Iran localities.

Elborz, Esfahan, Fars, Gilan, Markazi, Golestan, Mazandaran, Tehran.

#### Host plants.

Apocynaceae: *Nerium oleander*; Araliaceae: *Hedera canariensis*; Arecaceae: *Phoenix* sp.; Asparagaceae: *Asparagus officinalis*; Oleaceae: *Olea europaea*; Orchidaceae: *Maxillaria*, *longipetala*; Pinaceae: *Pinus* sp.; Tamaricaceae: *Tamarix* sp.

#### References.

Afchar (1937), Ben-Dov et al. (2013), Bodenheimer (1944), Borchsenius (1966), Davatchi (1946), Farahbakhsh (1961), Kaussari (1946, 1955), Kaussari and Farahbakhsh (1968), Kozár (1998), Kozár et al. (1996), Moghaddam (2004, 2010) and Seghatoleslami (1977).

#### Notes.

These are the first records of *Aspidiotus nerii* from the plant families Asparagaceae and Tamaricaceae.

### Aulacaspis phoenicis

(Green)

Diaspis phoenicis Green, 1922: 1014.

#### Iran localities.

Unknown.

#### Host plants.

Unknown plant.

#### References.

Kozár et al. (1996).

### Aulacaspis rosae

(Bouché)

Aspidiotus rosae Bouché, 1833: 53.

#### Iran localities.

Ardabil, Gilan, Mazandaran, Tehran.

#### Host plants.

Rosaceae: *Rosa* sp., *Rubus fruticosus*.

#### References.

Afchar (1937), Ben-Dov et al. (2013), Bodenheimer (1944), Borchsenius (1966), Davatchi (1946), Farahbakhsh (1961), Kaussari (1946, 1955), Kozár (1998), Kozár et al. (1996), Miller and Davidson (2005), Moghaddam (2004, 2010) and Seghatoleslami (1977).

### Balachowskiella salvadorae

Kaussari

Balachowskiella salvadorae Kaussari, 1955a: 230–232.

#### Iran localities.

Hormozgan, Sistan & Balouchestan.

#### Host plants.

Salvadoraceae: *Salvadora perisica*.

#### References.

Ben-Dov et al. (2013), Borchsenius (1966), Farahbakhsh (1961), Kaussari (1955a, 1957, 1964), Kozár et al. (1996), Seghatoleslami (1977), Moghaddam (2004) and Takagi and Moghaddam (2005).

### Carulaspis juniperi

(Bouché)

Carulaspis juniperi *Aspidiotus Juniperi* Bouché, 1851: 112.

#### Iran localities.

Unknown.

#### Host plants.

Unknown plant.

#### References.

Ben-Dov et al. (2013), Borchsenius (1966), Kozár (1998), Kozár et al. (1996) and Miller and Davidson (2005).

### Carulaspis minima

(Signoret)

Diaspis carueli Signoret, 1869d: 436. *Carulaspis caruelii* Borchsenius, 1966.

#### Iran localities.

Golestan, Mazandaran.

#### Host plants.

Cupressaceae: *Cupressus* sp., *Thuja orientalis*; Taxaceae: *Taxus* sp.

#### References.

Ben-Dov et al. (2013), Borchsenius (1966), Farahbakhsh (1961), Kaussari (1957), Kozár (1998), Kozár et al. (1996), Miller and Davidson (2005), Moghaddam (2004, 2010) and Seghatoleslami (1977).

#### Notes.

This is the first record of *Carulaspis minima* from the plant family Taxaceae.

### Carulaspis visci

(Schrank)

Coccus visci Schrank, 1781: 296. *Diaspis visci* Löw, 1872.

#### Iran localities.

Tehran.

#### Host plants.

Cuprassaceae: *Thuja* sp.

#### References.

Bodenheimer (1944).

### Chionaspis etrusca

Leonardi

Chionaspis etrusca Leonardi, 1908: 184–186.

#### Iran localities.

Azarbaijan -e Garbi, Azarbaijan -e Sharghi, Gilan, Kermanshah.

#### Host plants.

Tamaricaceae: *Tamarix* sp.

#### References.

Ben-Dov et al. (2013), Kozár (1998), Kozár et al. (1996), Moghaddam (2004, 2010) and Moghaddam and Tavakoli (2010).

### Chionaspis lepineyi

Balachowsky

Chionaspis lepineyi Balachowsky, 1928: 273–277. *Chionaspis parastigma* Balachowsky, 1954.

#### Iran localities.

Chaharmahal-Bakhtiari, Fars, Kohgilouyeh & Boyerahmad, Ilam, Lorestan.

#### Host plants.

Fagaceae: *Quercus* sp.

#### References.

Balachowsky (1954), Ben-Dov et al. (2013), Borchsenius (1966), Farahbakhsh (1961), Kaussari (1955, 1964), Kozár (1998), Kozár et al. (1996), Moghaddam (2004), Moghaddam and Tavakoli (2010) and Seghatoleslami (1977).

### Chionaspis salicis

(Linnaeus)

Coccus salicis Linnaeus, 1758: 456. *Chionaspis polypora* Borchsenius, 1949.

#### Iran localities.

Ardabil, Azarbaijan -e Garbi, Azarbaijan -e Sharghi, Gilan, Golestan, Hamadan, Ilam, Mazandaran, Semnan, Tehran.

#### Host plants.

Betulaceae *Alnus* sp.; Fagaceae: *Quercus* sp.; Oleaceae: *Fraxinus excelsior*; Salicaceae: *Populus nigra*, *Salix* sp.

#### References.

Afchar (1937), Ben-Dov et al. (2013), Bodenheimer (1944), Borchsenius (1966), Farahbakhsh (1961), Kaussari (1946, 1955), Kozár (1998), Kozár et al. (1996), Moghaddam (2004, 2010), Moghaddam and Tavakoli (2010) and Seghatoleslami (1977).

### Chlidaspis asiatica

(Archangelskaya)

Chionaspis asiatica Archangelskaya, 1930a: 93. *Chlidaspis prunorum* Borchsenius, 1949. *Phenacaspis prunorum* Borchsenius, 1939. *Tecaspis asiatica* Balachowsky, 1954. *Tecaspis prunorum* Balachowsky, 1954. *Voraspis adlei* Balachowsky & Kaussari, 1955.

#### Iran localities.

Chaharmahal-Bakhtiari, Elborz, Esfahan, Fars, Hamadan, Kerman, Kermanshah, Khorasan -e Shomali, Kordestan, Kohgilouyeh & Boyerahmad, Lorestan, Sistan & Balouchestan, Tehran, Yazd.

#### Host plants.

Rosaceae: *Malus domestica*, *Prunus amygdalus*, *Prunus armeniaca*, *Prunus domestica*, *Prunus lycioides*, *Prunus persica*, *Prunus scoparia*, *Prunus spinosa*, *Pyrus amygdaliformis* and *Pyrus communis*.

#### References.

Afchar (1937), Archangelskaya (1930), Ben-Dov et al. (2013), Bodenheimer (1944), Borchsenius (1966), Farahbakhsh (1961), Kaussari (1964), Kozár (1998), Kozár et al. (1996), Moghaddam (2004), Moghaddam and Tavakoli (2010) and Seghatoleslami (1977).

### ● Chortinaspis salavatiani

Balachowsky & Kaussari

Chortinaspis salavatiani Balachowsky & Kaussari, 1951: 2.

#### Iran localities.

Kermanshah, Sistan & Balouchestan.

#### Host plants.

Poaceae.

#### References.

Ben-Dov et al. (2013), Farahbakhsh (1961), Balachowsky and Kaussari (1951), Kaussari (1955), Kozár (1998), Kozár et al. (1996), Moghaddam (2004), Seghatoleslami (1977) and Torabi et al. (2010).

### Chrysomphalus dictyospermi

(Morgan)

Aspidiotus dictyospermi Morgan, 1889: 352.

#### Iran localities.

Gilan, Mazandaran, Tehran, Zanjan.

#### Host plants.

Apiaceae: *Actinolema eryngioides*; Arecaceae: *Howea forsteriana*, *Phoenix* sp.; Asparagaceae: *Beaucarnea recurvata*, *Dracaena* sp.; Buxaceae: *Buxus hyrcana*; Fabaceae: *Robinia* sp.; Moraceae: *Ficus benjamina*; Oleaceae: *Olea europea*; Pinaceae: *Pinus* sp.; Rutaceae: *Citrus sinensis*; Strelitziaceae: *Strelitzia alba*; Theaceae: *Camellia sinensis*.

#### References.

Afchar (1937), Ben-Dov et al. (2013), Bodenheimer (1944), Farahbakhsh (1961), Kaussari (1946, 1955), Kaussari and Farahbakhsh (1968), Kozár (1998), Kozár et al. (1996), Moghaddam (2004, 2010) and Seghatoleslami (1977).

#### Notes.

These are the first records of *Chrysomphalus dictyospermi* from the plant families Apiaceae and Asparagaceae.

### Chrysomphalus pinnulifer

(Maskell)

Diaspis pinnulifera Maskell, 1891: 4.

#### Iran localities.

Unknown.

#### Host plants.

Unknown plant.

#### References.

Kozár et al. (1996)

### ● Contigaspis davatchii

Kaussari

Contigaspis davatchii Kaussari, 1959: 131–134.

#### Iran localities.

Esfahan, Kohgilouyeh & Boyerahmad.

#### Host plants.

Amaranthaceae: *Noaea mucronata*; Asteraceae: *Cousinia* sp.

#### References.

Ben-Dov et al. (2013), Borchsenius (1966), Farahbakhsh (1961), Kaussari (1959, 1964), Kozár (1998), Kozár et al. (1996), Moghaddam (2004) and Seghatoleslami (1977).

#### Notes.

This is the first record of *Contigaspis davatchii* from the plant family Amaranthaceae.

### Contigaspis farsetiae

(Hall)

Coccomytilus farsetiae Hall, 1926: 23–24. *Eremohallaspis farsetiae* Balachowsky, 1954.
Artemisaspis farsetiae Borchsenius & Williams, 1963.

#### Iran localities.

Azarbaijan -e Garbi, Esfahan, Kerman, Khorasan -e Razavi, Sistan & Balouchestan.

#### Host plants.

Amaranthaceae: *Anabasis* sp., *Salsola* sp., *Seidlitzia rosmarinus*; Asteraceae: *Artemisia abrotanum*; Polygonaceae: *Atraphaxis spinosa*, *Calligonum comosum*; Zygophyllaceae: *Zygophyllum eurypterus*.

#### References.

Balachowsky (1954), Ben-Dov et al. (2013), Farahbakhsh (1961), Kaussari (1955), Kozár (1998), Kozár et al. (1996), Moghaddam (2004) and Seghatoleslami (1977).

### ● Contigaspis sarkissiani

(Kaussari & Balachowsky)

Paragadaspis sarkissiani Kaussari & Balachowsky in Balachowsky 1954: 162.

#### Iran localities.

Esfahan, Golestan, Kerman, Yazd.

#### Host plants.

Asteraceae: *Artemisia* sp.; Boraginaceae: *Heliotropium* sp.; Fabaceae: *Astragalus* sp.

#### References.

Balachowsky (1954), Ben-Dov et al. (2013), Borchsenius (1966), Farahbakhsh (1961), Kaussari (1955, 1964), Kozár (1998), Kozár et al. (1996), Moghaddam (2004, 2010) and Seghatoleslami (1977).

#### Notes.

This is the first record of *Contigaspis sarkissiani* from the plant family Fabaceae.

### Contigaspis zillae

(Hall)

Pinnaspis zillae Hall, 1923: 27–28.

#### Iran localities.

Azarbaijan -e Sharghi, Chaharmahal-Bakhtiari, Golestan, Hormozgan, Sistan & Balouchestan, Tehran.

#### Host plants.

Amaranthaceae: *Bassia prostrata*; Borajinaceae: *Heliotropium* sp.; Fabaceae: *Indigofera argentea*; Lamiaceae: *Thymus vulgaris*; Rosaceae: *Crataegus azarollus*.

#### References.

Ben-Dov et al. (2013), Borchsenius (1966), Borchsenius and Williams (1963), Farahbakhsh (1961), Kaussari (1955), Kozár (1998), Kozár et al. (1996), Moghaddam (2010) and Seghatoleslami (1977).

#### Notes.

These are the first records of *Contigaspis zillae* from the plant families Lamiaceae and Rosaceae.

### Cryptoparlatoreopsis halli

(Bodenheimer)

Aonidia halli Bodenheimer, 1929: 104–105.

#### Iran localities.

Kerman, Khorasan -e Shomali, Sistan & Balouchestan.

#### Host plants.

Lythraceae: *Punica granatum*; Tamaricaceae: *Tamarix* sp.

#### References.

Ben-Dov et al. (2013), Borchsenius (1966), Farahbakhsh (1961), Kaussari (1955, 1970), Kozár (1998), Kozár et al. (1996), Moghaddam (2004) and Seghatoleslami (1977).

#### Notes.

This is the first record of *Cryptoparlatoreopsis halli* from the plant family Lythraceae.

### Cryptoparlatoreopsis meccae

(Hall)

Targionia meccae Hall, 1927: 263–265.

#### Iran localities.

Sistan & Balouchestan.

#### Host plants.

Rhamnaceae: *Ziziphus spina-christi*.

#### References.

Ben-Dov et al. (2013), Borchsenius (1966), Farahbakhsh (1961), Kaussari (1955, 1970), Kozár (1998), Kozár et al. (1996) and Moghaddam (2004).

### Cryptoparlatoreopsis tlaiae

(Balachowsky)

Aonidia tlaiae Balachowsky, 1927: 200.

#### Iran localities.

Hormozgan, Sistan & Balouchestan.

#### Host plants.

Tamaricaceae: *Tamarix* sp.

#### References.

Ben-Dov et al. (2013), Farahbakhsh (1961), Kaussari (1955, 1970), Kozár et al. (1996), Moghaddam (2004) and Seghatoleslami (1977).

### Diaspidiotus armenicus

(Borchsenius)

Aspidiotus armenicus Borchsenius, 1935: 132.

#### Iran localities.

Azarbaijan -e Sharghi, Chaharmahal-Bakhtiari, Esfahan, Fars, Lorestan, Sistan & Balouchestan, Tehran.

#### Host plants.

Salicaceae: *Populus alba*, *Salix* sp.

#### References.

Balachowsky (1950a), Ben-Dov et al. (2013), Farahbakhsh (1961), Kaussari (1955), Kaussari and Farahbakhsh (1968), Kozár (1998), Kozár et al. (1996), Moghaddam (2004) and Seghatoleslami (1977).

### ● Diaspidiotus baiati

(Kaussari)

Quadraspidiotus baiati Kaussari, 1958: 232.

#### Iran localities.

Ardabil, Chaharmahal-Bakhtiari, Fars, Ilam, Khouzestan, Kohgilouyeh & Boyerahmad, Lorestan.

#### Host plants.

Ephedraceae: *Ephedra* sp.; Fabaceae: *Astragalus* sp.; Fagaceae: *Castanea sativa*; Thymelaeaceae: *Daphne angustifolia*.

#### References.

Ben-Dov et al. (2013), Borchsenius (1966), Kaussari (1958, 1964), Kaussari and Farahbakhsh (1968), Kozár (1998), Kozár et al. (1996), Moghaddam (2004), Moghaddam and Tavakoli (2010) and Seghatoleslami (1977).

#### Notes.

These are the first records of *Diaspidiotus baiati* from the plant families Ephedraceae and Fagaceae.

### Diaspidiotus caucasicus

(Borchsenius)

Aspidiotus caucasicus Borchsenius, 1935: 130.

#### Iran localities.

Fars, Kerman, Khorasan -e Shomali, Kohgilouyeh & Boyerahmad, Sistan & Balouchestan.

#### Host plants.

Salicaceae: *Salix* sp.

#### References.

Ben-Dov et al. (2013), Kaussari and Farahbakhsh (1968), Kozár (1998), Kozár et al. (1996), Moghaddam (2004) and Seghatoleslami (1977).

### Diaspidiotus cecconii

(Leonardi)

Hemiberlesia cecconii Leonardi, 1908: 188. *Quadraspidiotus cecconii* Balachowsky, 1950.

#### Iran localities.

Azarbaijan -e Garbi, Esfahan, Fars, Hamadan, Kerman, Kordestan.

#### Host plants.

Amaranthaceae: *Noaea mucronata*; Rosaceae: *Cotoneaster vulgaris*.

#### References.

Ben-Dov et al. (2013), Borchsenius (1966), Kaussari (1955), Kaussari and Farahbakhsh (1968), Kozár (1998), Kozár et al. (1996), Moghaddam (2004), Moghaddam and Tavakoli (2010) and Seghatoleslami (1977).

#### Notes.

This is the first record of *Diaspidiotus cocconii* from the plant family Rosaceae.

### Diaspidiotus elaeagni

(Borchsenius)

Aspidiotus elaeagni Borchsenius, 1939: 35.

#### Iran localities.

Kermanshah, Kohgilouyeh & Boyerahmad.

#### Host plants.

Fagaceae: *Quercus* sp.; Rosaceae: *Prunus* sp.

#### References.

Ben-Dov et al. (2013), Borchsenius (1966), Kozár (1998), Kozár et al. (1996) and Torabi et al. (2010).

#### Notes.

This is the first record of *Diaspidiotus elaeagni* from the plant family Fagaceae.

### Diaspidiotus farahbakhchi

Kaussari

Diaspidiotus farahbakhchi Kaussari, 1955a: 235.

#### Iran localities.

Mazandaran.

#### Host plants.

Fagaceae: *Quercus* sp.

#### References.

Ben-Dov et al. (2013), Borchsenius (1966), Farahbakhsh (1961), Kaussari (1955a, 1957, 1964), Kaussari and Farahbakhsh (1968), Kozár (1998), Kozár et al. (1996), Moghaddam (2004, 2010) and Seghatoleslami (1977).

### ● Diaspidiotus iranicus

Kaussari & Balachowsky

Diaspidiotus iranicus Kaussari & Balachowsky, 1953: 24.

#### Iran localities.

Esfahan, Fars, Kerman, Kermanshah, Khorasan -e Shomali, Kohgilouyeh & Boyerahmad, Sistan & Balouchestan.

#### Host plants.

Tamaricaceae: *Tamarix* sp.

#### References.

Ben-Dov et al. (2013), Borchsenius (1966), Farahbakhsh (1961), Kaussari and Balachowsky (1953), Kaussari (1955, 1964), Kaussari and Farahbakhsh (1968), Kozár (1998), Kozár et al. (1996), Moghaddam (2004) and Seghatoleslami (1977).

### Diaspidiotus kaussarii

Balachowsky

Diaspidiotus kaussari Balachowsky, 1950a: 494 (misspelling in Ben-Dov et al. 2013).

#### Iran localities.

Azarbaijan -e Sharghi, Kerman, Khorasan -e Shomali, Tehran.

#### Host plants.

Salicaceae: *Populus alba*, *Salix* sp.

#### References.

Balachowsky (1950a), Ben-Dov et al. (2013), Borchsenius (1966), Farahbakhsh (1961), Kaussari (1955, 1964), Kaussari and Farahbakhsh (1968), Kozár (1998), Kozár et al. (1996), Moghaddam (2004) and Seghatoleslami (1977).

### Diaspidiotus laperrinei

(Balachowsky)

Aspidiotus (Hemiberlesia) laperrinei Balachowsky, 1929: 314.

#### Iran localities.

Sistan & Balouchestan.

#### Host plants.

Polygonaceae: *Calligonum* sp.

#### References.

Ben-Dov et al. (2013), Farahbakhsh (1961), Kaussari (1955), Kaussari and Farahbakhsh (1968) and Kozár et al. (1996).

### * Diaspidiotus lenticularis

(Lindinger)

Aspidiotus lenticularis Lindinger, 1912: 149.

#### Iran localities.

Fars.

#### Host plants.

Moraceae: *Ficus carica*.

#### Notes.

This the first record of *Diaspidiotus lenticularis* from Iran, identified by B. Kaydan.

### Diaspidiotus ostreaeformis

(Curtis)

Aspidiotus ostreaeformis Curtis, 1843a: 805. *Quadraspidiotus ostreaeformis* MacGillivray, 1921.

#### Iran localities.

Chaharmahal-Bakhtiari, Mazandaran, Tehran.

#### Host plants.

Oleaceae: *Fraxinus excelsior*.

#### References.

Ben-Dov et al. (2013), Bodenheimer (1944), Farahbakhsh (1961), Kaussari (1955), Kozár (1998) and Kozár et al. (1996).

### Diaspidiotus perniciosus

(Comstock)

Aspidiotus perniciosus Comstock, 1881: 304.

#### Iran localities.

Ardabil, Gilan, Mazandaran.

#### Host plants.

Cucurbitaceae: *Citrullus vulgaris*; Rosaceae: *Malus domestica*, *Prunus* sp., *Rosa* sp.; Salicaceae: *Populus euramericana*, *Populus nigra*.

#### References.

Ben-Dov et al. (2013), CABI (1986), Kaussari and Farahbakhsh (1968), Kozár et al. (1996), Moghaddam (2004, 2010) and Seghatoleslami (1977).

#### Notes.

This is the first record of *Diaspidiotus pernicosus* from the plant family Cucurbitaceae.

### ● Diaspidiotus platychaetae

Takagi & Moghaddam

Diaspidiotus platychatae Takagi & Moghaddam, 2005: 54 (Misspelling of species name in Ben-Dov et al. 2013).

#### Iran localities.

Khouzestan.

#### Host plants.

Asteraceae: *Platychaeta mucroniflia*.

#### References.

Ben-Dov et al. (2013), Takagi and Moghaddam (2005).

### Diaspidiotus prunorum

(Laing)

Aspidiotus prunorum Laing, 1931: 99.

#### Iran localities.

Azarbaijan -e Garbi, Azarbaijan -e Sharghi, Chaharmahal-Bakhtiari, Esfahan, Fars, Golestan, Hamadan, Kerman, Kermanshah, Khorasan -e Shomali, Kohgilouyeh & Boyerahmad, Kordestan, Lorestan, Mazandaran, Tehran, Yazd.

#### Host plants.

Asteraceae: *Echinops ritro*; Rosaceae: *Malus domestica*, *Persica vulgaris*, *Prunus amygdalus*, *Prunus armeniaca*, *Prunus avium*, *Prunus cerasus*, *Prunus domestica*, *Prunus lycioides*, *Prunus persica*, *Amygdalus reuteri* *Prunus spinosa*, *Rosa* sp.; Tamaricaceae: *Tamarix* sp.

#### References.

Balachowsky (1950a), Ben-Dov et al. (2013), Borchsenius (1966), Dastgheyb-Beheshti et al. (1988), Farahbakhsh (1961), Kaussari (1946, 1955), Kaussari and Farahbakhsh (1968), Kozár (1998), Moghaddam (2004, 2010), Moghaddam and Tavakoli (2010) and Seghatoleslami (1977).

#### Notes.

This is the first record of *Diaspidiotus prunorum* from the plant family Tamaricaceae.

### Diaspidiotus pyri

(Lichtenstein)

Aspidiotus pyri Lichtenstein, 1881: lii. *Quadraspidiotus pyri* Lupo, 1948.

#### Iran localities.

Azarbaijan -e Garbi, Azarbaijan -e Sharghi, Khorasan -e Razavi.

#### Host plants.

Rosaceae: *Malus domestica*, *Prunus* sp., *Pyrus communis*.

#### References.

Ben-Dov et al. (2013), Farahbakhsh (1961), Kaussari (1946, 1955), Kaussari and Farahbakhsh (1968), Kozár (1998), Kozár et al. (1996), Moghaddam (2004) and Seghatoleslami (1977).

### Diaspidiotus slavonicus

(Green)

Targionia slavonica Green, 1934a: 95. *Quadraspidiotus slavonicus* Green, 1934. *Quadraspidiotus populi* Bodenheimer, 1943.

#### Iran localities.

Azarbaijan -e Sharghi, Chaharmahal-Bakhtiari, Elborz, Golestan, Khorasan -e Shomali, Lorestan, Markazi, Sistan & Balouchestan.

#### Host plants.

Oleaceae: *Fraxinus excelsior*; Salicaceae: *Populus euphratica*, *Salix caprica*.

#### References.

Ben-Dov et al. (2013), Bodenheimer (1944, 1944a), Farahbakhsh (1961), Kaussari (1955), Kaussari and Farahbakhsh (1968), Kozár (1998), Kozár et al. (1996), Moghaddam (2004, 2010), Moghaddam and Tavakoli (2010) and Seghatoleslami (1977).

#### Notes.

This is the first record of *Diaspidiotus slavonicus* from the plant family Oleaceae.

### Diaspidiotus transcaspiensis

(Marlatt)

Aspidiotus (Diaspidiotus) transcaspiensis Marlatt, 1908: 21.

#### Iran localities.

Esfahan, Golestan, Kerman.

#### Host plants.

Rosaceae: *Cotoneaster kotschyi*, *Prunus armeniaca*; Fagaceae: *Quercus castaneifolia*; Salicaceae: *Populus* sp.

#### References.

Ben-Dov et al. (2013), Farahbakhsh (1961), Kaussari (1955), Kaussari and Farahbakhsh (1968), Kozár (1998), Kozár et al. (1996), Moghaddam (2004, 2010) and Seghatoleslami (1977).

#### Notes.

This is the first record of *Diaspidiotus transcaspiensis* from the plant family Rosaceae.

### Diaspidiotus turanicus

(Borchsenius)

Aspidiotus turanicus Borchsenius, 1935: 131.

#### Iran localities.

Elborz, Kerman, Khorasan -e Shomali, Lorestan, Tehran, Zanjan.

#### Host plants.

Salicaceae: *Populus* sp., *Salix* sp.

#### References.

Ben-Dov et al. (2013), Farahbakhsh (1961), Kaussari (1955), Kaussari and Farahbakhsh (1968), Kozár et al. (1996), Moghaddam (2004), Moghaddam and Tavakoli (2010) and Seghatoleslami (1977).

### * Diaspidiotus wuenni

(Lindinger)

Aspidiotus wuenni Lindinger, 1923: 147.

#### Iran localities.

Kermanshah.

#### Host plants.

Fagaceae: *Quercus* sp.

#### Notes.

This is the first record of *Diaspidiotus wuenni* from Iran, identified by B. Kayadan.

### Diaspidiotus zonatus

(Frauenfeld)

Aspidiotus zonatus Frauenfeld, 1868: 888.

#### Iran localities.

Fars, Kohgilouyeh & Boyerahmad, Kordestan.

#### Host plants.

Fagaceae: *Quercus* sp.; Juglandaceae: *Juglans regia*; Moraceae: *Ficus carica*.

#### References.

Ben-Dov et al. (2013), Farahbakhsh (1961), Kaussari (1955), Kaussari and Farahbakhsh (1968), Moghaddam (2004), Kozár et al. (1996).

### Diaspis boisduvalii

Signoret

Diaspis boisduvalii Signoret, 1869b: 432–433.

#### Iran localities.

Gilan.

#### Host plants.

Arecaceae: *Chamaerops* sp.

#### References.

Ben-Dov et al. (2013), Farahbakhsh (1961), Kaussari (1957), Kozár (1998), Kozár et al. (1996), Moghaddam (2010) and Seghatoleslami (1977).

### ● Diaspis carmanica

Davatchi & Balachowsky

Diaspis carmanicus Davatchi & Balachowsky, 1956: 106–109.

#### Iran localities.

Fars, Kerman.

#### Host plants.

Anacardiaceae: *Pistacia khinjuk*.

#### References.

Ben-Dov et al. (2013), Davatchi and Balachowsky (1956), Farahbakhsh (1961), Kaussari (1964), Kozár (1998), Kozár et al. (1996) and Seghatoleslami (1977).

### Diaspis echinocacti

(Bouché)

Aspidiotus echinocacti Bouché, 1833: 53. *Diaspis calyptroides* Costa, 1829.

#### Iran localities.

Tehran.

#### Host plants.

Cactaceae: *Opuntia* sp.

#### References.

Ben-Dov et al. (2013) and Bodenheimer (1944).

### Diaspis syriaca

Lindinger

Diaspis syriaca Lindinger, 1912: 264.

#### Iran localities.

Kermanshah.

#### Host plants.

Anacardiaceae: *Pistacia khinjuk*.

#### References.

Ben-Dov et al. (2013), Bodenheimer (1944), Borchsenius (1966), Farahbakhsh (1961), Kaussari (1955), Kozár (1998) and Kozár et al. (1996).

### Duplachionaspis graminella

(Borchsenius)

Chionaspis graminella Borchsenius, 1949: 348.

#### Iran localities.

Ilam, Kerman.

#### Host plants.

Poaceae: *Phragmites australis*.

#### References.

Balachowsky (1954), Ben-Dov et al. (2013), Borchsenius (1966), Farahbakhsh (1961), Kaussari (1955), Kozár (1998), Kozár et al. (1996) and Moghaddam and Tavakoli (2010).

### Duplachionaspis natalensis

(Maskell)

Chionaspis spartinae natalensis Maskell, 1896: 390–391. *Duplachionaspis stanotophri* Hall, 1946.

#### Iran localities.

Kerman, Sistan & Balouchestan.

#### Host plants.

Poaceae: *Cynodon dactylon*, *Phragmites* sp.

#### References.

Balachowsky (1954), Ben-Dov et al. (2013), Borchsenius (1966) and Kozár et al. (1996).

### Duplachionaspis noaeae

(Hall)

Chionaspis noaeae Hall, 1925: 13–14.

#### Iran localities.

Ardabil, Chaharmahal-Bakhtiari, Hormozgan, Lorestan.

#### Host plants.

Amaranthaceae: *Noaea mucronata*.

#### References.

Moghaddam and Tavakoli (2010).

### Dynaspidiotus abieticola

(Koroneos)

Aspidiotus abieticola Koroneos, 1934: 9.

#### Iran localities.

Unknown.

#### Host plants.

Pinaceae.

#### References.

Ben-Dov et al. (2013) and Kaussari (1954).

### Dynaspidiotus abietis

(Schrank)

Coccus abietis Schrank, 1781: 48.

#### Iran localities.

Mazandaran.

#### Host plants.

Pinaceae: *Pinus* sp.

#### References.

Ben-Dov et al. (2013), Kaussari (1954), Abai (1995) and Moghaddam (2004, 2010).

### ● Dynaspidiotus amygdalicola

(Borchsenius)

Diaspidiotus amygdalicola Borchsenius, 1952: 261.

#### Iran localities.

Fars, Ilam, Kerman, Kohgilouyeh & Boyerahmad, Lorestan.

#### Host plants.

Rosaceae: *Prunus lycioides*, *Prunus reuteri*, *Prunus scoparia*.

#### References.

Ben-Dov et al. (2013), Borchsenius (1952, 1966), Farahbakhsh (1961), Kaussari (1955, 1964), Komosinska (1969), Kozár (1998), Moghaddam (2004), Moghaddam and Tavakoli (2010) and Seghatoleslami (1977).

### Dynaspidiotus atlanticus

(Balachowsky)

Hemiberlesia atlantica Balachowsky, 1928: 125.

#### Iran localities.

Unknown.

#### Host plants.

Araliaceae: *Hedera helix*.

#### References.

Ben-Dov et al. (2013) and Kaussari (1954).

### Dynaspidiotus britannicus

(Newstead)

Aspidiotus britannicus Newstead, 1898: 93.

#### Iran localities.

Unknown.

#### Host plants.

Aquifoliaceae: *Ilex* sp.; Araliaceae: *Hedera helix*; Asparagaceae: *Ruscus aculeatus*;Buxaceae: *Buxus* sp.; Lauraceae: *Laurus nobilis*.

#### References.

Ben-Dov et al. (2013) and Kaussari (1954).

### Dynaspidiotus ephedrarum

(Lindinger)

Aspidiotus ephedrarum Lindinger, 1912: 139. *Abgrallaspis ephedrarum* Balachowsky, 1948.

#### Iran localities.

Fars, Hormozgan, Kerman, Semnan, Yazd.

#### Host plants.

Ephedraceae: *Ephedra* sp.

#### References.

Ben-Dov et al. (2013), Farahbakhsh (1961), Kaussari (1955), Kozár (1998), Kozár et al. (1996), Moghaddam (2004) and Seghatoleslami (1977).

### Dynaspidiotus ericarum

(Goux)

Aspidiotus ericarum Goux, 1937: 345.

#### Iran localities.

Unknown.

#### Host plants.

Anacardiaceae: *Pistacia lentiscus*; Ericaceae: *Arbutus unedo*; Myrtaceae: *Myrtus communis*.

#### References.

Ben-Dov et al. (2013) and Kaussari (1954).

### ● Dynaspidiotus medicus

Kaussari

Dynaspidiotus medicus Kaussari, 1956: 102.

#### Iran localities.

Kerman, Sistan & Balouchestan.

#### Host plants.

Cruciferae: *Sameraria*; Rutaceae: *Haplophyllum* sp.; Solanaceae: *Withania somnifera*.

#### References.

Ben-Dov et al. (2013), Borchsenius (1966), Farahbakhsh (1961), Kaussari (1956, 1964), Kozár (1998), Kozár et al. (1996) and Seghatoleslami (1977).

#### Notes.

This is the first record of *Dynaspidiotus medicus* from the plant family Solanaceae.

### ● Dynaspidiotus spartii

Kaussari

Dynaspidiotus spartii Kaussari, 1954: 82.

#### Iran localities.

Gilan, Kermanshah.

#### Host plants.

Fabaceae: *Spartium junceum*.

#### References.

Ben-Dov et al. (2013), Borchsenius (1966), Farahbakhsh (1961), Kaussari (1954, 1957, 1964), Kaussari and Farahbakhsh (1968), Kozár (1998), Kozár et al. (1996), Moghaddam (2004, 2010) and Seghatoleslami (1977).

### Dynaspidiotus tener

(Bazarov & Shmelev)

Ephedraspis tenera Bazarov & Shmelev, 1967: 60.

#### Iran localities.

Golestan.

#### Host plants.

Ephedraceae: *Ephedra* sp.

#### References.

Ben-Dov et al. (2013) and Moghaddam (2000a, 2004, 2010).

### Epidiaspis gennadii

(Leonardi)

Diaspis gennadii Leonardi, 1898: 115.

#### Iran localities.

Ghazvin.

#### Host plants.

Anacardiaceae: *Pistacia vera*.

#### References.

Ben-Dov et al. (2013), Davatchi (1958), Farahbakhsh (1961), Kozár (1998) and Kozár et al. (1996).

### Epidiaspis leperii

(Signoret)

Diaspis leperii Signoret, 1869b: 437–438.

#### Iran localities.

Tehran.

#### Host plants.

Rosaceae.

#### References.

Ben-Dov et al. (2013), Balachowsky (1954), Borchsenius (1966), Farahbakhsh (1961), Kaussari (1955), Kozár (1998), Kozár et al. (1996) and Miller and Davidson (2005).

### Epidiaspis salicis

(Bodenheimer)

Thymaspis salicis Bodenheimer, 1944a: 94–95.

#### Iran localities.

Chaharmahal-Bakhtiari.

#### Host plants.

Salicaceae: *Salix* sp.

#### References.

Ben-Dov et al. (2013), Bodenheimer (1944a), Borchsenius (1966), Farahbakhsh (1961), Kaussari (1955, 1964), Kozár (1998), Kozár et al. (1996) and Seghatoleslami (1977).

### Fiorinia distinctissima

(Newstead)

Parlatoria distinctissima Newstead, 1896: 133–134. *Fiorinia afchari* Bodenheimer, 1944.

#### Iran localities.

Hormozgan, Kerman, Sistan & Balouchestan.

#### Host plants.

Apocynaceae: *Nerium oleander*, *Periploca aphylla*; Capparaceae: *Capparis decidua*; Solanaceae: *Withania somnifera*.

#### References.

Ben-Dov et al. (2013), Bodenheimer (1944), Borchsenius (1966), Farahbakhsh (1961), Kaussari (1955, 1964), Kozár (1998), Kozár et al. (1996) and Seghatoleslami (1977).

#### Notes.

These are the first records of *Fiorinia distinctissima* from the plant families Capparaceae and Solanaceae.

### Fiorinia phoenicis

Balachowsky

Fiorinia phoenicis Balachowsky, 1967: 771–775.

#### Iran localities.

Fars, Hormozgan, Kerman, Sistan & Balouchestan.

#### Host plants.

Arecaceae: *Phoenix dactylifera*.

#### References.

Balachowsky (1967), Ben-Dov et al. (2013), Kozár (1998) and Takagi and Moghaddam (2005).

### * Fiorinia proboscidaria

Green

Fiorinia proboscidaria Green, 1900: 256.

#### Iran localities.

Sistan & Balouchestan.

#### Host plants.

Unknown plant.

#### Notes.

This is the first record of *Fiorinia proboscidaria* from Iran, identified by M. Moghaddam.

### Froggattiella penicillata

(Green)

Odonaspis penicillata Green, 1905: 346.

#### Iran localities.

Gilan.

#### Host plants.

Poaceae: *Bambusa* sp.

#### References.

Ben-Dov (1988), Ben-Dov et al. (2013), Borchsenius (1966), Farahbakhsh (1961), Kaussari (1955, 1970), Kozár (1998), Kozár et al. (1996), Moghaddam (2010) and Seghatoleslami (1977).

### ● Gonaspidiotus kaussarii

(Balachowsky)

Abgrallaspis kaussarii Balachowsky, 1959: 211.

#### Iran localities.

Semnan, Tehran.

#### Host plants.

Unknown plant.

#### References.

Balachowsky (1959), Ben-Dov et al. (2013), Farahbakhsh (1961), Kaussari and Farahbakhsh (1968), Kozár (1998), Kozár et al. (1996) and Seghatoleslami (1977).

### Hemiberlesia lataniae

(Signoret)

Aspidiotus lataniae Signoret, 1869: 124.

#### Iran localities.

Gilan, Mazandaran, Sistan & Balouchestan.

#### Host plants.

Buxaceae: *Buxus hyrcana*; Fabaceae: *Inga edulis*; Lauraceae: *Persea americana*.

#### References.

Ben-Dov et al. (2013), Kaussari and Farahbakhsh (1968), Kozár (1998), Kozár et al. (1996) and Moghaddam (2004, 2010).

### Hemiberlesia rapax

(Comstock)

Aspidiotus rapax Comstock, 1881: 307. *Aspidiotus camelliae* Signoret, 1869 (nomen nudum). *Hemiberlesia camelliae* Leonardi, 1897 (nomen nudum).

#### Iran localities.

Gilan, Mazandaran, Sistan & Balouchestan.

#### Host plants.

Celastraceae: *Euonymus japonicus*; Fabaceae: *Robinia* sp.; Moraceae: *Ficus* sp.; Oleaceae: *Olea europea*.

#### References.

Ben-Dov et al. (2013), Davatchi (1946), Farahbakhsh (1961), Kaussari (1955), Kaussari and Farahbakhsh (1968), Kozár (1998), Kozár et al. (1996), Moghaddam (2004, 2010) and Seghatoleslami (1977).

### Koroneaspis aegilopos

(Koroneos)

Lepidosaphes aegilopos Koroneos, 1934: 74–75.

#### Iran localities.

Fars, Kermanshah, Kohgilouyeh & Boyerahmad.

#### Host plants.

Fagaceae: *Quercus* sp.

#### References.

Balachowsky (1954), Ben-Dov et al. (2013), Borchsenius (1966), Farahbakhsh (1961), Kaussari (1955), Kozár (1998) and Kozár et al. (1996).

### * Koroneaspis lonicerae

Borchsenius

Koroneaspis lonicerae Borchsenius, 1949a: 343–344.

#### Iran localities.

Chaharmahal-Bakhtiari, Fars, Kerman, Kohgilouyeh & Boyerahmad.

#### Host plants.

Caprifoliaceae: *Lonicera* sp.

#### Notes.

This is the first record of *Koroneaspis lonicerae* from Iran, identified by M. Moghaddam.

### Kuwanaspis howardi

(Cooley)

Chionaspis howardi Cooley, 1898: 88–89.

#### Iran localities.

Mazandaran.

#### Host plants.

Poaceae: *Bambusa* sp.

#### References.

Moghaddam (2003, 2004, 2010).

### Lepidosaphes afganensis

Borchsenius

Lepidosaphes afganensis Borchsenius, 1962: 861.

#### Iran localities.

Unknown.

#### Host plants.

Unknown plant.

#### References.

Ben-Dov et al. (2013), Kozár et al. (1996).

### Lepidosaphes beckii

(Newman)

Coccus beckii Newman, 1869: 217–218.

#### Iran localities.

Mazandaran.

#### Host plants.

Buxaceae: *Buxus hyrcana*; Rutaceae: *Citrus sinensis*; Theaceae: *Camellia sinensis*.

#### References.

Afchar (1937), Ben-Dov et al. (2013), Bodenheimer (1944), Farahbakhsh (1961), Kaussari (1946, 1955), Kozár (1998), Kozár et al. (1996), Moghaddam (2004, 2010) and Seghatoleslami (1977).

#### Notes.

This is the first record of *Lepidosaphes beckii* from the plant family Buxaceae.

### Lepidosaphes belutchistana

Balachowsky

Lepidosaphes belutchistanus Balachowsky, 1954: 75–77. *Mytilaspis belutchistanus* Borchsenius, 1963.

#### Iran localities.

Hormozgan, Sistan & Balouchestan, Kerman.

#### Host plants.

Acanthaceae: *Avicenniae officinalis*; Arecaceae: *Nannorrhops ritchiana*; Apocynaceae: *Nerium oleander*, *Periploca aphylla*; Fabaceae: *Acacia* sp., *Prosopis spicigera*.

#### References.

Balachowsky (1954), Ben-Dov et al. (2013), Borchsenius (1966), Kaussari (1955, 1964), Farahbakhsh (1961), Kozár (1998), Kozár et al. (1996), Takagi and Moghaddam (2005) and Seghatoleslami (1977).

#### Notes.

These are the first records of *Lepidosaphes belutchistana* from the plant families Acanthaceae and Arecaceae.

### Lepidosaphes conchiformis

(Gmelin)

Coccus conchiformis Gmelin, 1790: 2221. *Lepidosaphes ficus* Fernald, 1903. *Lepidosaphes minima* Fernald, 1903. *Mytilaspis conchiformis* Signoret, 1870.

#### Iran localities.

Esfahan, Fars, Ghazvin, Golestan, Kerman, Khorasan -e Jonoubi, Sistan & Balouchestan, Tehran.

#### Host plants.

Elaeagnaceae: *Elaeagnus angustifolia*; Juglandaceae: *Juglans regia*; Moraceae: *Ficus carica*; Ulmaceae: *Ulmus campestris*.

#### References.

Ben-Dov et al. (2013), Farahbakhsh (1961), Kaussari (1946, 1955), Kozár (1998), Kozár et al. (1996), Moghaddam (2004) and Seghatoleslami (1977).

### Lepidosaphes gloverii

(Packard)

Aspidiotus gloverii Packard, 1869: 527.

#### Iran localities.

Gilan, Mazandaran.

#### Host plants.

Rutaceae: *Citrus* spp.

#### References.

Afchar (1937), Ben-Dov et al. (2013), Bodenheimer (1944), Farahbakhsh (1961), Kaussari (1946, 1955), Kozár (1998), Kozár et al. (1996), Moghaddam (2004, 2010) and Seghatoleslami (1977).

### Lepidosaphes granati

Koroneos

Lepidosaphes conchiformis granati Koroneos, 1934: 72–73.

#### Iran localities.

Esfahan, Khorasan -e Jonoubi.

#### Host plants.

Elaeagnaceae: *Eleagnus* sp.

#### References.

Ben-Dov et al. (2013), Farahbakhsh (1961), Kaussari (1955), Kozár (1998) and Kozár et al. (1996).

### Lepidosaphes janguai

Balachowsky

Lepidosaphes janguai Balachowsky, 1954: 82–83.

#### Iran localities.

Sistan & Balouchestan.

#### Host plants.

Tamaricaceae: *Tamarix* sp.

#### References.

Ben-Dov et al. (2013), Kaussari (1964) and Kozár et al. (1996).

### Lepidosaphes juniperi

Lindinger

Lepidosaphes juniperi Lindinger, 1912: 188. *Mytilococcus juniperi* Bodenheimer, 1943.

#### Iran localities.

Hamadan, Tehran.

#### Host plants.

Cupressaceae: *Juniperus communis*, *Thuja orientalis*.

#### References.

Ben-Dov et al. (2013), Bodenheimer (1944), Borchsenius (1966), Farahbakhsh (1961), Kaussari (1946, 1955), Kozár (1998), Kozár et al. (1996) and Seghatoleslami (1977).

### Lepidosaphes malicola

Borchsenius

Lepidosaphes malicola Borchsenius, 1947: 142.

#### Iran localities.

Azarbaijan -e Garbi, Azarbaijan -e Sharghi, Chaharmahal-Bakhtiari, Elborz, Esfahan, Fars, Gilan, Hamadan, Kerman, Khorasan -e Razavi, Khorasan -e Shomali, Kohgilouyeh & Boyerahmad, Kordestan, Lorestan, Mazandaran, Tehran.

#### Host plants.

Fabaceae: *Astragalus* sp., *Cercis siliquastrum*; Juglandaceae: *Juglans regia*; Lythraceae: *Punica granatum*; Oleaceae: *Fraxinus excelsior*; Rosaceae: *Cotoneaster vulgaris*, *Prunus armeniaca*, *Prunus persica*, *Prunus* sp.; Salicaceae: *Populus nigra*; Thymelaeaceae: *Daphne angustifolia*; Ulmaceae: *Ulmus* sp.

#### References.

Ben-Dov et al. (2013), Borchsenius (1966), Farahbakhsh (1961), Kaussari (1955), Kozár (1998), Kozár et al. (1996), Moghaddam (2004, 2010), Moghaddam and Tavakoli (2010) and Seghatoleslami (1977).

#### Notes.

These are the first records of *Lepidosaphes malicola* from the plant families Lythraceae, Thymelaeaceae and Ulmaceae.

### Lepidosaphes newsteadi

(Šulc)

Mytilaspis newsteadi Šulc, 1895: 8–12.

#### Iran localities.

Kerman.

#### Host plants.

Apocynaceae: *Nerium oleander*.

#### References.

Ben-Dov et al. (2013), Danzig and Pellizzari (1998) and Kozár (1998).

#### Notes.

This is the first record of *Lepidosaphes newsteadi* from the plant family Apocynaceae.

### Lepidosaphes pallida

(Maskell)

Mytilaspis pallida Maskell, 1895: 46. *Insulaspis maskelli* Borchsenius, 1963.

#### Iran localities.

Gilan, Golestan, Sistan & Balouchestan.

#### Host plants.

Acanthaceae: *Avicennia officinalis*; Apocynaceae: *Nerium* sp.; Cupressaceae: *Juniperus communis*; Pinaceae: *Pinus* sp.; Rosaceae: *Mespilus germanica*;Taxodiaceae: *Cryptomeria* sp.

#### References.

Ben-Dov et al. (2013), Kozár (1998), Kozár et al. (1996), Moghaddam (2010) and Seghatoleslami (1977).

#### Notes.

These are the first records of *Lepidosaphes pallida* from the plant families Acanthaceae, Apocynaceae and Rosaceae.

### Lepidosaphes pallidula

(Williams)

Insulaspis pallidula Williams, 1969: 114.

#### Iran localities.

Sistan & Balouchestan.

#### Host plants.

Myrtaceae: *Psidium guajava*; Solanaceae: *Solanum melongena*.

#### References.

Takagi and Moghaddam (2005).

#### Notes.

This is the first record of *Lepidosaphes pallidula* from the plant family Solanaceae.

### Lepidosaphes pinnaeformis

(Bouché)

Aspidiotus pinnaeformis Bouché, 1851: 111.

#### Iran localities.

Unknown.

#### Host plants.

Unknown plant.

#### References.

Archangelskaya (1937) and Ben-Dov et al. (2013).

### Lepidosaphes pistaciae

Archangelskaya

Lepidosaphes pistaciae Archangelskaya, 1930: 91. *Mytilococcus pistaciae* Bodenheimer, 1943. *Pistaciaspis pistaciae* Borchsenius, 1963. *Pistaciapis pistacicola* Borchsenius, 1963.

#### Iran localities.

Azarbaijan -e Garbi, Esfahan, Fars, Ghazvin, Ilam, Kerman, Kermanshah, Khorasan -e Razvi, Kohgilouyeh & Boyerahmad, Kordestan, Lorestan, Sistan & Balouchestan, Yazd.

#### Host plants.

Anacardiaceae: *Pistacia khinjuk*, *Pistacia mutica*, *Pistacia vera*.

#### References.

Archangelskaya (1937), Ben-Dov et al. (2013), Borchsenius (1966), Farahbakhsh (1961), Kaussari (1955), Kozár et al. (1996), Moghaddam (2004), Moghaddam and Tavakoli (2010) and Seghatoleslami (1977).

### Lepidosaphes turanica

Archangelskaya

Lepidosaphes turanica Archangelskaya, 1937: 75. *Mytilaspis turanica* (Archangelskaya, 1937).

#### Iran localities.

Unknown.

#### Host plants.

Uknown plant.

#### References.

Ben-Dov et al. (2013), Borchsenius (1966) and Kozár et al. (1996).

### Lepidosaphes ulmi

(Linnaeus)

Coccus ulmi Linnaeus, 1758: 455.

#### Iran localities.

Ardabil, Azarbaijan -e Garbi, Azarbaijan -e Sharghi, Fars, Gilan, Golestan, Lorestan, Markazi, Mazandaran, Tehran.

#### Host plants.

Aceraceae: *Acer* sp.; Betulaceae: *Alnus glutinosa*, *Corylus avellana*; Fabaceae: *Cercis siliquastrum*, *Spartium junceum*; Juglandaceae: *Juglans regia*; Oleaceae: *Fraxinus excelsior*; Rosaceae: *Crataegus ambigua*, *Mespilus germanica*, *Prunus* sp., *Rosa* sp.; Salicaceae: *Populus nigra*, *Salix* sp.; Ulmaceae: *Ulmus carpinifolia*.

#### References.

Afchar (1937), Ben-Dov et al. (2013), Borchsenius (1966), Farahbakhsh (1961), Kaussari (1946, 1955), Kozár (1998), Kozár et al. (1996), Moghaddam (2004, 2010), Moghaddam and Tavakoli (2010) and Seghatoleslami (1977).

### Leucaspis lowi

Colvée

Leucaspis lowi Colvée, 1882: 10–12.

#### Iran localities.

Unknown.

#### Host plants.

Uknown plant.

#### References.

Ben-Dov et al. (2013), Kozár (1998) and Kozár et al. (1996).

### Leucaspis pusilla

Löw

Leucaspis pusilla Löw, 1883: 3–5.

#### Iran localities.

Elborz, Fars, Golestan, Mazandaran, Tehran.

#### Host plants.

Pinaceae: *Pinus* sp.

#### References.

Ben-Dov et al. (2013), Farahbakhsh (1961), Kaussari (1955, 1970), Kozár (1998), Kozár et al. (1996), Moghaddam (2004, 2010) and Seghatoleslami (1977).

### Leucaspis riccae

Targioni Tozzetti

Leucaspis riccae *Leucaspis Riccae* Targioni Tozzetti, 1881: 160.

#### Iran localities.

Kermanshah, Zanjan.

#### Host plants.

Apocynaceae: *Nerium oleander*; Oleaceae: *Olea europea*.

#### References.

Ben-Dov et al. (2013), Farahbakhsh (1961), Kaussari (1955, 1970), Kozár et al. (1996), Moghaddam and Tavakoli (2010) and Seghatoleslami (1977).

### Lopholeucaspis japonica

(Cockerell)

Leucaspis japonicus Cockerell, 1897: 53.

#### Iran localities.

Gilan, Hormozgan, Mazandaran, Sistan & Balouchestan.

#### Host plants.

Aceraceae: *Acer insigne*; Betulaceae: *Corylus avellana*; Buxaceae: *Buxus hyrcana*; Caprifoliaceae: *Lonicera caprifolium*; Celastraceae: *Euonymus japonicus*; Fabaceae: *Robinia* sp.; Magnoliaceae: *Magnolia grandiflora*; Moraceae: *Ficus bengalensis*, *Ficus carica*, *Ficus riligiosa*, *Morus alba*; Rosaceae: *Cydonia oblonga*, *Mespilus germanica*, *Rosa* sp.

#### References.

Ben-Dov et al. (2013), Kaussari (1970), Kaussari and Farahbakhsh (1968), Kozár et al. (1996), Miller and Davidson (2005), Moghaddam (2004, 2010) and Seghatoleslami (1977).

#### Notes.

This is the first record of *Lopholeucaspis japonica* from the plant family Buxaceae.

### Melanaspis inopinata

(Leonardi)

Aonidiella inopinata Leonardi, 1913: 63.

#### Iran localities.

Azarbaijan -e Sharghi, Bushehr, Fars, Kerman, Kermanshah, Kordestan, Kohgilouyeh & Boyerahmad, Lorestan, Yazd.

#### Host plants.

Aceraceae: *Acer cinerascens*; Brassicaceae: *Anabasis* sp.; Anacardiaceae: *Pistacia khinjuk*, *Pistacia mutica*; Euphorbiaceae: *Ricinus communis*; Fabaceae: *Acacia* sp., *Astragalus* sp.; Fagaceae: *Quercus* sp.; Juglandaceae: *Juglans regia*; Oleaceae: *Fraxinus excelsior*; Rosaceae: *Malus domestica*, *Prunus avium*, *Pyrus* sp.; Salicaceae: *Salix* sp.

#### References.

Ben-Dov et al. (2013), Farahbakhsh (1961), Kaussari (1955), Kaussari and Farahbakhsh (1968), Kozár (1998), Kozár et al. (1996), Moghaddam (2004), Moghaddam and Tavakoli (2010) and Seghatoleslami (1977).

#### Notes.

These are the first records of *Melanaspis inopinata* from the plant families Brassicaceae, Euphorbiaceae and Fagaceae.

### ● Melanaspis louristanus

Balachowsky & Kaussari

Melanaspis louristanus Balachowsky & Kaussari, 1953: 28.

#### Iran localities.

Chaharmahal-Bakhtiari, Fars, Ilam, Kermanshah, Kordestan, Khouzestan, Kohgilouyeh & Boyerahmad, Lorestan.

#### Host plants.

Fagaceae: *Quercus* sp.

#### References.

Balachowsky and Kaussari (1953), Ben-Dov et al. (2013), Borchsenius (1966), Farahbakhsh (1961), Kaussari (1964, 1955), Kaussari and Farahbakhsh (1968), Kozár (1998), Kozár et al. (1996), Moghaddam (2004), Moghaddam and Tavakoli (2010) and Seghatoleslami (1977).

### Mercetaspis bicuspis

(Hall)

Lepidosaphes bicuspis Hall, 1923: 22–23. *Nilotaspis bicuspis* Balachowsky, 1954.

#### Iran localities.

Kerman, Sistan & Balouchestan.

#### Host plants.

Amaranthaceae: *Suaeda nudiflora*; Sapindaceae: *Stocksia brahuica*; Tamaricaceae: *Tamarix* sp.

#### References.

Ben-Dov et al. (2013), Farahbakhsh (1961), Kaussari (1955), Kozár (1998), Kozár et al. (1996) and Seghatoleslami (1977).

#### Notes.

These are the first records of *Mercetaspis bicuspis* from the plant families Amaranthaceae and Sapindaceae.

### Mercetaspis calligoni

(Borchsenius)

Haplaspis calligoni Borchsenius, 1949: 736.

#### Iran localities.

Kerman.

#### Host plants.

Polygonaceae: *Atraphaxis spinosa*, *Calligonum denticulatum*.

#### References.

Ben-Dov et al. (2013), Farahbakhsh (1961), Kaussari (1955), Kozár (1998) and Kozár et al. (1996).

### Mercetaspis halli

(Green)

Lepidosaphes (Coccomytilus) halli Green, 1923: 63. *Nilotaspis halli* Ferris, 1941.

#### Iran localities.

Ardabil, Azarbaijan -e Garbi, Azarbaijan -e Sharghi, Chaharmahal-Bakhtiari, Esfahan, Fars, Hamadan, Kerman, Kermanshah, Kohgilouyeh & Boyerahmad, Lorestan, Sistan & Balouchestan, Yazd.

#### Host plants.

Anacardiaceae: *Pistacia khinjuk*; Fabaceae: *Astragalus* sp., *Ebenus stellata*; Rosaceae: *Amygdalus communis*, *Amygdalus lycioides*, *Amygdalus persica*, *Amygdalus reuteri*, *Amygdalus scoparia*, *Armeniaca vulgaris*, *Prunus amygdalus*, *Prunus armeniaca*, *Prunus domestica*, *Prunus persica*, *Prunus reuteri*, *Prunus sorparia*, *Prunus spinosa*.

#### References.

Balachowsky (1954), Ben-Dov et al. (2013), Borchsenius (1966), Farahbakhsh (1961), Kaussari (1946, 1955), Kozár (1998), Kozár et al. (1996), Miller and Davidson (2005), Moghaddam (2004), Moghaddam and Tavakoli (2010) and Seghatoleslami (1977).

#### Notes.

This is the first record of *Mercetaspis halli* from the plant family Anacardiaceae.

### Mercetaspis isis

(Hall)

Coccomytilus isis Hall, 1923: 21–22. *Nilotaspis isis* Hall, 1923.

#### Iran localities.

Esfahan, Hormozgan, Kerman.

#### Host plants.

Tamaricaceae: *Tamarix* sp.

#### References.

Balachowsky (1954), Ben-Dov et al. (2013), Farahbakhsh (1961), Kaussari (1955), Kozár (1998), Kozár et al. (1996), Moghaddam (2004), Seghatoleslami (1977) and Takagi and Moghaddam (2005).

### Mercetaspis sureyanus

(Bodenheimer)

Coccomytilus sureyanus Bodenheimer, 1941: 66.

#### Iran localities.

Golestan.

#### Host plants.

Fabaceae: *Astragalus* sp.

#### References.

Moghaddam (2001, 2010)

### Mongrovaspis quadrispinosa

(Green)

Leucaspis quadrispinosa Green, 1934: 110–111.

#### Iran localities.

Sistan & Balouchestan.

#### Host plants.

Acanthaceae: *Avicennia officinalis*.

#### References.

Takagi and Moghaddam (2005).

### Mycetaspis personata

(Comstock)

Aspidiotus personatus Comstock, 1883: 66.

#### Iran localities.

Unknown.

#### Host plants.

Uknown plant.

#### References.

Kozár et al. (1996).

### Oceanaspidiotus spinosus

(Comstock)

Aspidiotus spinous Comstock, 1883: 70.

#### Iran localities.

Sistan & Balouchestan.

#### Host plants.

Solanaceae: *Solanum melongena*.

#### References.

Ben-Dov et al. (2013) and Takagi and Moghaddam (2005).

### Odonaspis panici

Hall

Odonaspis panici Hall, 1926: 26.

#### Iran localities.

Hormozgan.

#### Host plants.

Poaceae.

#### References.

Ben-Dov et al. (2013), Borchsenius (1966), Ben-Dov (1988), Farahbakhsh (1961), Kaussari (1970), Kozár (1998), Kozár et al. (1996), Moghaddam (2004) and Seghatoleslami (1977).

### Odonaspis secreta

(Cockerell)

Aspidiotus secretus Cockerell, 1896: 20.

#### Iran localities.

Gilan.

#### Host plants.

Poaceae: *Bambusa* sp.

#### References.

Ben-Dov et al. (2013), Farahbakhsh (1961), Kaussari (1955, 1970) and Kozár et al. (1996).

### Parlagena buxi

(Takahashi)

Gymnaspis buxi Takahashi, 1936: 220–222.

#### Iran localities.

Unknown.

#### Host plants.

Unknown plant.

#### References.

Ben-Dov et al. (2013) and Kozár et al. (1996).

### ● Parlagena mckenziei

Balachowsky

Parlagena mckenziei *Parlagena McKenzei* Balachowsky, 1950: 18–20.

#### Iran localities.

Bushehr, Esfahan, Hormozgan, Ilam, Khouzestan, Sistan & Balouchestan.

#### Host plants.

Fabaceae: *Prosopis stephaniana*; Tamaricaceae: *Tamarix* sp.

#### References.

Balachowsky (1950), Ben-Dov et al. (2013), Borchsenius (1966), Farahbakhsh (1961), Kaussari (1964, 1970), Kozár (1998), Kozár et al. (1996), Moghaddam (2004), Moghaddam and Tavakoli (2010) and Seghatoleslami (1977).

#### Notes.

This is the first record of *Parlagena mckenziei* from the plant family Fabaceae.

### ● Parlagena remaudierei

Kaussari

Parlagena remaudierei *Parlagena Remaudierei* Kaussari, 1955a: 234–235.

#### Iran localities.

Esfahan, Kerman, Kermanshah, Sistan & Balouchestan.

#### Host plants.

Convolvulaceae: *Convolvulus* sp.; Lythraceae: *Punica granatum*; Zygophyllaceae: *Zygophyllum eurypeterum*.

#### References.

Ben-Dov et al. (2013), Borchsenius (1966), Farahbakhsh (1961), Kaussari (1955a, 1957, 1964, 1970), Kozár (1998), Kozár et al. (1996), Moghaddam (2004), Moghaddam and Tavakoli (2010) and Seghatoleslami (1977).

#### Notes.

These are the first records of *Parlagena remaudierei* from the plant families Convolvulaceae and Lythraceae.

### Parlatoreopsis chinensis

(Marlatt)

Parlatoria chinensis Marlatt, 1908: 30–32.

#### Iran localities.

Unknown.

#### Host plants.

Unknown plant.

#### References.

Kozár et al. (1996).

### Parlatoreopsis longispina

(Newstead)

Chionaspis longispina Newstead, 1911: 88–89.

#### Iran localities.

Hormozgan, Ilam, Kerman, Kermanshah, Kordestan.

#### Host plants.

Lythraceae: *Punica granatum*; Moraceae: *Ficus carica*; Polygonaceae: *Calligonum denticulatum*; Rhamnaceae: *Ziziphus spina-christi*; Rosaceae: *Prunus spinosa*, *Rosa* sp.; Tamaricaceae: *Tamarix* sp.; Zygophyllaceae: *Zygophyllum* sp.

#### References.

Ben-Dov et al. (2013), Farahbakhsh (1961), Kaussari (1955, 1970), Kozár (1998), Kozár et al. (1996), Moghaddam (2004), Moghaddam and Tavakoli (2010) and Seghatoleslami (1977).

#### Notes.

This is the first record of *Parlatoreopsis longispina* from the plant family Tamaricaceae.

### Parlatoria asiatica

Borchsenius

Parlatoria asiatica Borchsenius, 1949b: 341.

#### Iran localities.

Kerman.

#### Host plants.

Ephedraceae: *Ephedra* sp.

#### References.

Ben-Dov et al. (2013), Borchsenius (1966), Kaussari (1970), Kozár (1998), Kozár et al. (1996), Moghaddam (2004, 2010) and Seghatoleslami (1977).

### Parlatoria blanchardi

(Targioni Tozzetti)

Aonidia blanchardi Targioni Tozzetti, 1892: 69–82.

#### Iran localities.

Bushehr, Fars, Hormozgan, Ilam, Kerman, Sistan & Balouchestan, Yazd.

#### Host plants.

Arecaceae: *Chamaerops humilis*, *Nannorrhops ritchiana*, *Phoenix dactylifera*.

#### References.

Afchar (1937), Ben-Dov et al. (2013), Bodenheimer (1944), Borchsenius (1966), Carpenter and Elmer (1978), Farahbakhsh (1961), Kaussari (1946, 1955, 1970), Kozár (1998), Kozár et al. (1996), Miller and Davidson (2005), Moghaddam (2004), Moghaddam and Tavakoli (2010), Seghatoleslami (1977).

### Parlatoria camelliae

Comstock

Parlatoria pergandii camelliae Comstock, 1883: 114.

#### Iran localities.

Unknown.

#### Host plants.

Unknown plant.

#### References.

Ben-Dov et al. (2013), Kozár (1998) and Kozár et al. (1996).

### Parlatoria crypta

McKenzie

Parlatoria crypta McKenzie, 1943: 156. *Parlatoria morrisoni*, McKenzie, 1943.

#### Iran localities.

Bushehr, Fars, Hormozgan, Kerman, Kermanshah, Khouzestan, Sistan & Balouchestan.

#### Host plants.

Anacardiaceae: *Mangifera indica*; Apocynaceae: *Calotropis procera*; *Nerium oleander*; Arecaceae: *Phoenix dactylifera*; Asparagaceae: *Yucca baccata*; Boraginaceae: *Cordia* sp.; Convolvulaceae: *Ipomoea* sp.; Fabaceae: *Acacia* sp., *Albizzia lebbek*, *Glycyrrhiza glabra*, *Indigofera argentea*;: Lythraceae: *Lawsonia inermis*; Meliaceae: *Melia azadirachta*; Moraceae: *Ficus bengalensis*; *Morus alba*; Myrtaceae: *Myrtus communis*; Oleaceae: *Fraxinus excelsior*, *Olea europea*; Polygonaceae: *Calligonum comosum*; Rhamnaceae: *Ziziphus spina-christi*; Rosaceae: *Malus domestica*, *Pyrus communis*, *Rosa* sp.; Rutaceae: *Citrus limonia*.

#### References.

Balachowsky (1953), Ben-Dov et al. (2013), Bodenheimer (1944), Borchsenius (1966), Farahbakhsh (1961), Kaussari (1955, 1970), Kozár (1998), Matile-Ferrero (1984), Kozár et al. (1996), Moghaddam (2004) and Seghatoleslami (1977).

#### Notes.

These are the first records of *Parlatoria crypta* from the plant families Asclepiadaceae, Asparagaceae, Boraginaceae, Convolvulaceae, Fabaceae, Lythraceae, Polygonaceae and Rhamnaceae.

### Parlatoria ephedrae

(Lindinger)

Parlatorea ephedrae Lindinger, 1911: 129. *Archangelskaia ephedrae* Bodenheimer, 1951.

#### Iran localities.

Esfahan, Golestan, Kerman, Sistan & Balouchestan.

#### Host plants.

Ephedraceae: *Ephedra* sp.

#### References.

Ben-Dov et al. (2013), Bodenheimer (1944), Borchsenius (1966), Farahbakhsh (1961), Kaussari (1955, 1970), Kozár (1998), Kozár et al. (1996), Moghaddam (2004) and Seghatoleslami (1977).

### Parlatoria oleae

(Colvée)

Diaspis oleae Colvée, 1880: 40. *Syngenaspis oleae* MacGillivray, 1921.

#### Iran localities.

Ardebil, Azarbaijan -e Garbi, Azarbaijan -e Sharghi, Elborz, Esfahan, Fars, Gilan, Golestan, Kerman, Kermanshah, Khorasan -e Razvi, Khorasan -e Shomali, Kohgilouyeh & Boyerahmad, Lorestan, Mazandaran, Sistan & Balouchestan, Tehran, Yazd.

#### Host plants.

Anacardiaceae: *Pistacia mutica*, *Rhus coriaria*; Apiaceae: *Heracleum persicum*; Apocynaceae: *Nerium oleander*; Asparagaceae: *Asparagus plumosus*; Berberidaceae: *Berberis vulgaris*; Boraginaceae: *Cordia myxa*; Ebenaceae: *Diospyrus kaki*; Fabaceae: *Astragalus* sp., *Gleditsia* sp., *Robinia pseudo-acacia*; Fagaceae: *Quercus* sp.; Juglandaceae: *Juglans regia*; Lythraceae: *Punica granatum*; Malvaceae: *Hibiscus syriacus*; Oleaceae: *Fraxinus excelsior*, *Jasminum officinalis*, *Olea europea*;Rhamnaceae: *Rhamnus* sp.; Rosaceae: *Cotoneaster vulgaris*, *Crataegus ambigua*, *Cydonia vulgaris*, *Malus domestica*, *Mespilus germanica*, *Persica vulgaris*, *Prunus amygdalus*, *Prunus armenica*, *Prunus avium*, *Prunus caspica*, *Pyrus communis*, *Pyrus persica*, *Rosa damascena*; Rutaceae: *Citrus* sp.; Salicaceae: *Populus tremula*; Ulmaceae: *Ulmus campestris*.

#### References.

Afchar (1937), Ben-Dov et al. (2013), Bodenheimer (1944), Borchsenius (1966), Davatchi (1946), Farahbakhsh (1961), Kaussari (1946, 1955, 1970), Kozár (1998), Kozár et al. (1996), Moghaddam (2004, 2010), Moghaddam and Tavakoli (2010) and Seghatoleslami (1977).

#### Notes.

These are the first records of *Parlatoria oleae* from the plant families Apiaceae and Fagaceae.

### Parlatoria pergandii

Comstock

Parlatoria pergandii Comstock, 1881: 327–328. *Syngenaspis pergandii* MacGillivray, 1921.

#### Iran localities.

Gilan, Mazandaran.

#### Host plants.

Rosaceae: *Prunus laurocerasus*; Rutaceae: *Citrus bigaradia*, *Citrus sinensis*.

#### References.

Ben-Dov et al. (2013), Farahbakhsh (1961), Kaussari (1946, 1955, 1970), Kozár (1998), Kozár et al. (1996), Moghaddam (2004, 2010) and Seghatoleslami (1977).

### Parlatoria proteus

(Curtis)

Parlatoria proteus *Aspidiotus Proteus* Curtis, 1843: 676.

#### Iran localities.

Gilan, Mazandaran.

#### Host plants.

Theaceae: *Camellia* sp.

#### References.

Ben-Dov et al. (2013), Farahbakhsh (1961), Kaussari (1955, 1970) and Kozár et al. (1996).

### Parlatoria theae

Cockerell

Parlatoria theae Cockerell, 1896: 21.

#### Iran localities.

Gilan, Mazandaran.

#### Host plants.

Rosaceae: *Prunus* sp., *Rosa* sp.; Theaceae: *Camellia sinensis*.

#### References.

Ben-Dov et al. (2013), Farahbakhsh (1961), Kaussari (1955, 1970), Kozár (1998), Kozár et al. (1996), Moghaddam (2004, 2010) and Seghatoleslami (1977).

### Parlatoria ziziphi

(Lucas)

Coccus ziziphi Lucas, 1853: xxix.

#### Iran localities.

Gilan, Golestan, Mazandaran.

#### Host plants.

Rutaceae: *Citrus* sp.

#### References.

Afchar (1937), Ben-Dov et al. (2013), Bodenheimer (1944), Borchsenius (1966), Farahbakhsh (1961), Kaussari (1946, 1955, 1970), Kozár (1998), Kozár et al. (1996), Moghaddam (2004, 2010) and Seghatoleslami (1977).

### Pinnaspis aspidistrae

(Signoret)

Chionaspis aspidistrae Signoret, 1869b: 443.

#### Iran localities.

Gilan, Sistan & Balouchestan.

#### Host plants.

Arecaceae: *Chamaerops humilis*; Rhamnaceae: *Ziziphus spina-christi*.

#### References.

Ben-Dov et al. (2013), Borchsenius (1966), Farahbakhsh (1961), Kaussari (1957), Seghatoleslami (1977), Kozár (1998), Kozár et al. (1996), Moghaddam (2004, 2010) and Seghatoleslami (1977).

### Pinnaspis strachani

(Cooley)

Hemichionaspis minor strachani Cooley, 1899: 54–55.

#### Iran localities.

Sistan & Balouchestan.

#### Host plants.

Fabaceae: *Acacia* sp.; Malvaceae: *Gossypium arboreum*; Rhamnaceae: *Ziziphus spina-christi*.

#### References.

Moghaddam (2000).

### Prodiaspis tamaricicola

(Malenotti)

Adiscodiaspis tamaricicola Malenotti, 1916: 313–315. *Adiscodiaspis tamaricicola* Malenotti, 1916. *Rungaspidiotus tamaricicola* Balachowsky, 1953.

#### Iran localities.

Azarbaijan -e Garbi, Bushehr, Esfahan, Fars, Khouzestan, Khorasan -e Razavi, Khorasan -e Shomali, Sistan & Balouchestan, Yazd.

#### Host plants.

Tamaricaceae: *Tamarix* sp.

#### References.

Ben-Dov et al. (2013), Farahbakhsh (1961), Kaussari (1955, 1970), Moghaddam (2004), Moghaddam and Tavakoli (2010) and Seghatoleslami (1977).

### Pseudaonidia duplex

(Cockerell)

Aspidiotus duplex Cockerell, 1896: 20.

#### Iran localities.

Sistan & Balouchestan.

#### Host plants.

Anacardiaceae: *Mangifera indica*.

#### References.

Moghaddam (2002).

### Pseudaulacaspis pentagona

(Targioni Tozzetti)

Diaspis pentagona Targioni Tozzetti, 1886: 1.

#### Iran localities.

Ardabil, Gilan, Golestan, Mazandaran.

#### Host plants.

Actinidiaceae: *Actinida chinensis*; Juglandaceae: *Juglans regia*; Moraceae: *Morus alba*; Pinaceae: *Pinus* sp.; Rosaceae: *Prunus persica*; Rutaceae: *Citrus bigaradia*; Salicaceae: *Salix* sp.

#### References.

Ben-Dov et al. (2013), Kozár (1998), Kozár et al. (1996), Moghaddam (2004, 2010) and Seghatoleslami (1977).

#### Notes.

This is the first record of *Pseudaulacaspis pentagona* from the plant family Pinaceae.

### Pseudotargionia glandulosa

(Newstead)

Aonidia glandulosa Newstead, 1911: 103.

#### Iran localities.

Sistan & Balouchestan.

#### Host plants.

Fabaceae: *Acacia farnesiana*.

#### References.

Ben-Dov et al. (2013), Moghaddam (2001, 2004), Kaussari (1970) and Kozár (1998).

### ● Pseudotargionia orientalis

Balachowsky & Kaussari

Pseudotargionia orientalis Balachowsky & Kaussari, 1951: 3.

#### Iran localities.

Sistan & Balouchestan.

#### Host plants.

Bignoniaceae: *Catalpa speciosa*; Fabaceae: *Acacia farnesiana*; Sapindaceae: *Stocksia brahuica*.

#### References.

Balachowsky and Kaussari (1951), Ben-Dov et al. (2013), Farahbakhsh (1961), Kaussari (1955, 1964), Kozár (1998), Kozár et al. (1996), Moghaddam (2004) and Seghatoleslami (1977).

#### Notes.

These are the first records of *Pseudotargionia orientalis* from the plant families Bignoniaceae and Fabaceae.

### Rhizaspidiotus canariensis

(Lindinger)

Aspidiotus canariensis Lindinger, 1911a: 12.

#### Iran localities.

Golestan.

#### Host plants.

Asteraceae: *Achillea* sp.

#### References.

Ben-Dov et al. (2013), Kozár (1998), Kozár et al. (1996) and Moghaddam (2004, 2010).

### Rhizaspidiotus secretus

(Borchsenius)

Arundaspis secretus Borchsenius, 1949b: 738.

#### Iran localities.

Unknown

#### Host plants.

Poaceae.

#### References.

Kozár et al. (1996).

### ● Rungaspis avicenniae

Takagi & Moghaddam

Rungaspis avicenniae Takagi & Moghaddam, 2005: 53.

#### Iran localities.

Hormozgan, Sistan & Balouchestan.

#### Host plants.

Acanthaceae: *Avicennia officinalis*.

#### References.

Ben-Dov et al. (2013) and Takagi and Moghaddam (2005).

### Rungaspis capparidis

(Bodenheimer)

Diaspis capparidis Bodenheimer, 1929: 108. *Rungaspis trabuti* Balachowsky, 1949.

#### Iran localities.

Hormozgan.

#### Host plants.

Acanthaceae: *Avicennia officinalis*.

#### References.

Balachowsky (1958), Ben-Dov et al. (2013), Farahbakhsh (1961), Kaussari (1955), Kozár (1998), Kozár et al. (1996), Moghaddam (2004), Seghatoleslami (1977) and Takagi and Moghaddam (2005).

### Rungaspis macrolobis

Kaussari

Rungaspis macrolobis Kaussari, 1958: 229.

#### Iran localities.

Esfahan, Hormozgan, Kerman, Sistan & Balouchestan.

#### Host plants.

Amaranthaceae: *Anabasis aphylla*; Asclepiadaceae: *Calotropis procera*; Fabaceae: *Robinia* sp.

#### References.

Ben-Dov (1980), Ben-Dov et al. (2013), Farahbakhsh (1961), Kaussari (1958, 1964), Kaussari and Farahbakhsh (1968), Kozár (1998), Kozár et al. (1996), Moghaddam (2004) and Seghatoleslami (1977).

#### Note.

These are the first records of *Rungaspis macrolobis* from the plant families Asclepiadaceae and Fabaceae.

### Salicicola archangelskyae

(Lindinger)

Leucaspis archangelskyae Lindinger, 1929: 113. *Suturaspis archangelskyae* Borchsenius, 1937.

#### Iran localities.

Azarbaijan -e Sharghi, Bushehr, Elborz, Esfahan, Fars, Gilan, Kerman, Kermanshah, Kohgilouyeh & Boyerahmad, Lorestan, Tehran.

#### Host plants.

Aceraceae: *Acer cinerascens*; Oleaceae: *Fraxinus excelsior*, *Olea europea*; Rosaceae: *Prunus domestica*, *Prunus reuteri*, *Pyrus communis*; Thymelaeaceae: *Daphne angustifolia*; Ulmaceae: *Celtis australis*.

#### References.

Ben-Dov et al. (2013), Borchsenius (1966), Farahbakhsh (1961), Kaussari (1946, 1955, 1970), Kozár (1998), Kozár et al. (1996), Moghaddam (2004, 2010) and Seghatoleslami (1977) and Takagi and Moghaddam (2005).

#### Note.

These are the first records of *Salicicola archangelskyae* from the plant families Aceraceae and Ulmaceae.

### Salicicola davatchii

Balachowsky& Kaussari

Salicicola davatchi Balachowsky & Kaussari, 1951: 6–7. *Suturaspis davatchi* Borchsenius, 1966. (misspelling in Ben-Dov et al. 2013).

#### Iran localities.

Azarbaijan -e Garbi, Fars, Hormozgan, Ilam, Kerman, Kermanshah, Kohgilouyeh & Boyerahmad, Lorestan, Sistan & Balouchestan.

#### Hosts.

Anacardiaceae: *Pistacia khinjuk*; Moraceae: *Ficus carica*, *Pistacia mutica*; Rosaceae: *Prunus lycioides*.

#### References.

Balachowsky and Kaussari (1951), Ben-Dov et al. (2013), Borchsenius (1966), Farahbakhsh (1961), Kaussari (1955, 1964, 1970), Kozár (1998), Kozár et al. (1996), Moghaddam (2004), Moghaddam and Tavakoli (2010) and Seghatoleslami (1977).

#### Note.

This is the first record of *Salicicola davatchi* from the plant family Rosaceae.

### Salicicola kermanensis

(Lindinger)

Leucaspis (Salicicola) kermanensis Lindinger, 1905: 253–254. *Leucaspis kermanensis* Lindinger, 1905.

#### Iran localities.

Azarbaijan -e Garbi, Azarbaijan -e Sharghi, Chaharmahal-Bakhtiari, Elborz, Esfahan, Fars, Golestan, Kerman, Khorasan -e Shomali, Khouzestan, Kohgilouyeh & Boyerahmad, Kordestan, Lorestan, Markazi, Sistan & Balouchestan, Yazd.

#### Host plants.

Salicaceae: *Populus alba*, *Populus euphratica*, *Populus nigra*, *Salix babylonica*.

#### References.

Ben-Dov et al. (2013), Bodenheimer (1944), Farahbakhsh (1961), Kaussari (1946, 1955, 1970), Kozár (1998), Kozár et al. (1996), Moghaddam (2004, 2010), Moghaddam and Tavakoli (2010) and Seghatoleslami (1977).

### Salicicola pistaciae

(Lindinger)

Leucaspis (Euleucaspis) pistaciae Lindinger, 1906: 40.

#### Iran localities.

Kerman, Kermanshah.

#### Host plants.

Anacardiaceae: *Pistacia mutica*.

#### References.

Afchar (1937), Ben-Dov et al. (2013), Bodenheimer (1944), Borchsenius (1966), Farahbakhsh (1961), Kaussari (1955, 1970), Kozár (1998), Kozár et al. (1996), Moghaddam (2004) and Seghatoleslami (1977).

### Targionia anabasidis

(Borchsenius)

Pseudomelanaspis minima Borchsenius, 1952: 262. *Targaspidiotus anabsidis* Borchsenius, 1952.

#### Iran localities.

Hormozgan.

#### Host plants.

Amaranthaceae: *Anabasis aphylla*.

#### References.

Ben-Dov et al. (2013), Borchsenius (1952), Danzig (1993), Farahbakhsh (1961), Kaussari (1955), Kozár (1998) and Kozár et al. (1996).

### Targionia arthrophyti

(Archangelskaya)

Aspidiotus (Aonidiella) arthrophyti Archangelskaya, 1931: 83. *Schizotargionia arthrophyti* Balachowsky.

#### Iran localities.

Yazd.

#### Host plants.

Amaranthaceae: *Haloxylon* sp.

#### References.

Bazarov and Shmelev (1971), Ben-Dov et al. (2013), Borchsenius (1966), Kozár (1998), Kozár et al. (1996) and Moghaddam (2004).

### ● Targionia balachowskyi

(Kaussari)

Schizotargionia balachowskyi Kaussari, 1952: 182.

#### Iran localities.

Esfahan, Sistan & Balouchestan.

#### Host plants.

Moraceae: *Haloxylon* sp.; Tamaricaceae: *Tamarix* sp.

#### References.

Ben-Dov et al. (2013), Borchsenius (1966), Farahbakhsh (1961), Kaussari (1952, 1955, 1970), Kozár (1998), Kozár et al. (1996), Moghaddam (2004) and Seghatoleslami (1977).

#### Note.

This is the first record of *Targionia balachowskyi* from the plant family Amaranthaceae.

### Targionia haloxyloni

Hall

Targionia haloxyloni Hall, 1926: 27.

#### Iran localities.

Lorestan.

#### Host plants.

Fabaceae: *Astragalus* sp.; Thymelaeaceae: *Daphne* sp.

#### References.

Ben-Dov et al. (2013), Kaussari (1970), Kozár (1998), Kozár et al. (1996) and Moghaddam and Tavakoli (2010).

### Targionia nigra

Signoret

Targionia nigra Signoret, 1870: 106.

#### Iran localities.

Hormozgan, Sistan & Balouchestan.

#### Host plants.

Amaranthaceae: *Anabasis* sp.; Resedaceae: *Ochradenus rostratus*, *Ochradenus socotrnus*.

#### References.

Balachowsky (1951), Ben-Dov et al. (2013), Borchsenius (1966), Farahbakhsh (1961), Kaussari (1955, 1970), Kozár (1998), Kozár et al. (1996), Moghaddam (2004) and Seghatoleslami (1977).

### Targionia porifera

(Borchsenius)

Rhizaspidiotus porifera Borchsenius, 1949a: 350. *Fisanotargionia quadrilobata* Kaussari & Balachowsky, 1953.

#### Iran localities.

Esfahan, Fars, Hormozgan, Kerman, Yazd.

#### Host plants.

Amaranthaceae: *Anabasis* sp., *Seidlitzia schweinfurthii*; Asteraceae: *Artemisia* sp.; Zygophyllaceae: *Zygophyllum eurypeterum*.

#### References.

Ben-Dov et al. (2013), Borchsenius (1966), Farahbakhsh (1961), Kaussari and Balachowsky (1953), Kaussari (1957, 1964, 1970), Kozár (1998), Kozár et al. (1996), Moghaddam (2004) and Seghatoleslami (1977).

#### Note.

These are the first records of *Targionia porifera* from the plant families Amaranthaceae and Asteraceae.

### Targionia vitis

(Signoret)

Aspidiotus vitis Signoret, 1876: lii.

#### Iran localities.

Gilan, Golestan.

#### Host plants.

Fagaceae: *Quercus* sp.

#### References.

Ben-Dov et al. (2013), Danzig (1993), Farahbakhsh (1961), Kaussari (1955, 1970), Kozár (1998), Kozár et al. (1996), Moghaddam (2004, 2010) and Seghatoleslami (1977).

### Torosapis cedricola

(Balachowsky & Alkan)

Acanthomytilus cedricola Balachowsky & Alkan, 1956: 320.

#### Iran localities.

Gilan, Kerman.

#### Host plants.

Cupressaceae: *Cupressus* sp.

#### References.

Moghaddam (2004, 2010).

### ● Torosapis farsianus

(Balachowsky & Kaussari)

Acanthomytilus farsianus Balachowsky & Kaussari, 1955: 238.

#### Iran localities.

Fars.

#### Host plants.

Cupressaceae: *Cupressus* sp.

#### References.

Balachowsky and Alkan (1956), Balachowsky and Kaussari (1955), Ben-Dov et al. (2013), Borchsenius (1966), Danzig and Pellizzari (1998), Farahbakhsh (1961), Kaussari (1964), Kozár (1998), Kozár et al. (1996), Kozár and Walter (1985), Moghaddam (2004, 2010), Takagi (1970) and Ülgentürk and Kozár (2011).

### Unaspis euonymi

(Comstock)

Chionaspis euonymi Comstock, 1881: 313–314.

#### Iran localities.

Gilan.

#### Host plants.

Celastraceae: *Euonymus japonicus*.

#### References.

Balachowsky (1954), Ben-Dov et al. (2013), Borchsenius (1966), Kozár et al. (1996) and Seghatoleslami (1977).

## Family ERIOCOCCIDAE

### Acanthococcus araucariae

(Maskell)

Eriococcus araucariae Maskell, 1879: 218.

#### Iran localities.

Unknown.

#### Host plants.

Araucariaceae: *Araucaria* sp.

#### References.

Ben-Dov et al. (2013), Farahbakhsh (1961) and Kozár et al. (1996).

### Cryptococcus fagisuga

Lindinger

Kermes fagi ; Lindinger, 1936: 444.

#### Iran localities.

Gilan, Mazandaran.

#### Host plants.

Fagaceae: *Fagus orientalis*.

#### References.

Ben-Dov et al. (2013), Kozár et al. (1996) and Moghaddam (2009, 2010).

### ● Eriococcus abaii

(Danzig)

Acanthococcus abaii Danzig, 1990: 373–376.

#### Iran localities.

Kerman, Sistan & Balouchestan.

#### Host plants.

Amaranthaceae: *Haloxylon aphylla*.

#### References.

Ben-Dov et al. (2013), Kozár et al. (1996), Miller and Gimpel (2000), Moghaddam (2009), Moghaddam and Tavakoli (2010) and Tang and Hao (1995).

### * Eriococcus cingulatus

Kiritchenko

Eriococcus cingulatus Kiritchenko, 1940: 131–133.

#### Iran localities.

Azarbaijan -e Sharghi.

#### Host plants.

Unknown Plant.

#### Note.

This is the first record of *Eriococcus cingulatus* from Iran, identified by F. Kozár.

### Eriococcus costatus

(Danzig)

Acanthococcus costatus Danzig, 1975: 43.

#### Iran localities.

Kermanshah.

#### Host plants.

Ulmaceae: *Ulmus* sp.

#### References.

Ben-Dov et al. (2013) and Torabi et al. (2010).

### Eriococcus isacanthus

(Danzig)

Acanthococcus isacanthus Danzig, 1975: 45.

#### Iran localities.

Kermanshah.

#### Host plants.

Ulmaceae: *Ulmus* sp.

#### References.

Ben-Dov et al. (2013) and Torabi et al. (2010).

### Eriococcus kondarensis

(Borchsenius)

Rhizococcus kondarensis Borchsenius, 1949: 353–354.

#### Iran localities.

Golestan, Khorasan -e Shomali.

#### Host plants.

Poaceae.

#### References.

Moghaddam (2001, 2009, 2010)

### * Eriococcus pamiricus

(Bazarov)

Acanthococcus pamiricus Bazarov, 1968: 73.

#### Iran localities.

Sistan & Balouchestan.

#### Host plants.

Unknown plant.

#### Note.

This is the first record of *Eriococcus pamiricus* in Iran, identified by M. Moghaddam.

### * Eriococcus reynei

Schmutterer

Eriococcus reynei Schmutterer, 1952: 414–417.

#### Iran localities.

Ghazvin.

#### Host plants.

Lamiaceae: *Thymus vulgaris*.

#### Notes.

This is the first record of *Eriococcus reynei* in Iran, identified by F. Kozár.

### * Eriococcus sanguinairensis

Goux

Eriococcus sanguinairensis Goux, 1993: 68–69.

#### Iran localities.

Ardabil.

#### Host plants.

Unknown plant.

#### Note.

This is the first record of *Eriococcus sanguinairensis* in Iran, identified by F. Kozár.

### * Eriococcus saxidesertus

(Borchsenius)

Acanthococcus saxidesertus Borchsenius, 1949: 343.

#### Iran localities.

Ardabil.

#### Host plants.

Unknown plant.

#### Note.

This is the first record of *Eriococcus saxidesertus* in Iran, identified by F. Kozár.

### Eriococcus spurius

(Modeer)

Coccus spurius Modeer, 1778: 43. *Gossyparia spuria* Cockerell, 1899.

#### Iran localities.

Esfahan, Fars, Golestan, Hamadan, Kerman, Markazi.

#### Host plants.

Ulmaceae: *Ulmus carpenifolia*.

#### References.

Ben-Dov et al. (2013), Bodenheimer (1944), Farahbakhsh (1961), Hoy (1963), Kaussari (1946, 1957), Kozár (1998), Kozár et al. (1996), Moghaddam (2009, 2010) and Moghaddam and Tavakoli (2010).

### Neoacanthococcus tamaricicola

Borchsenius

Neoacanthococcus tamaricicola Borchsenius, 1948: 502–503.

#### Iran localities.

Khouzestan.

#### Host plants.

Tamaricaceae: *Tamarix* sp.

#### References.

Moghaddam (2009).

### Pseudochermes fraxini

(Kaltenbach)

Chermes fraxini Kaltenbach, 1860: 259. *Fonscolombea fraxini* Cockerell, 1899.

#### Iran localities.

Tehran.

#### Host plants.

Oleaceae: *Fraxinus excelsior*.

#### References.

Ben-Dov et al. (2013), Bodenheimer (1944), Hoy (1963) and Kozár et al. (1996).

## Family KERMESIDAE

### Kermes quercus

(Linnaeus)

Coccus quercus Linnaeus, 1758: 455. *Kermococcus quercus* Henriksen, 1921: 308.

#### Iran localities.

Ilam, Kermanshah.

#### Host plants.

Fagaceae: *Quercus* sp.

#### References.

Ben-Dov et al. (2013), Farahbakhsh (1961), Kozár et al. (1996), Moghaddam (2009) and Moghaddam and Tavakoli (2010).

### Nidularia balachowskii

Bodenheimer

Nidularia balachowskii Bodenheimer, 1941: 78–80.

#### Iran localities.

Kermanshah, Lorestan.

#### Host plants.

Fagaceae: *Quercus* sp.

#### References.

Ben-Dov et al. (2013) and Bodenheimer (1941, 1944).

## Family MARGARODIDAE

### ● Porphyrophora chelodonta

Vahedi

Porphyrophora chelodonta Vahedi in Vahedi and Hodgson 2007: 40.

#### Iran localities.

Kermanshah.

#### Host plants.

Unknown plant.

#### References.

Ben-Dov et al. (2013) and Vahedi and Hodgson (2007).

### Porphyrophora cynodontis

(Archangelskaya)

Margarodes cynodontis Archangelskaya, 1935: 15.

#### Iran localities.

Azarbaijan -e Sharghi.

#### Host plants.

Poaceae: *Cynodon dactylon*.

#### References.

Ben-Dov et al. (2013), Kozár et al. (1996), Moghaddam (2009), Torabi et al. (2010), Vahedi (2002) and Vahedi and Hodgson (2007).

### Porphyrophora hamelii

Brandt

Porphyrophora hamelii Brandt in Brandt and Ratzeburg 1833: 356.

#### Iran localities.

Ardabil, Hamadan.

#### Host plants.

Poaceae.

#### References.

Ben-Dov et al. (2013), Kaussari (1957), Kozár et al. (1996), Vahedi (2002) and Vahedi and Hodgson (2007).

### ● Porphyrophora jashenkoi

Vahedi

Porphyrophora jashenkoi Vahedi in Vahedi and Hodgson 2007: 68.

#### Iran localities.

Kermanshah.

#### Host plants.

Poaceae: *Triticum vulgare*.

#### References.

Ben-Dov et al. (2013) and Vahedi and Hodgson (2007).

### Porphyrophora medicaginis

Jashenko

Porphyrophora medicaginis Jashenko, 1994: 22.

#### Iran localities.

Azarbaijan -e Sharghi, Kerman.

#### Host plants.

Fabaceae: *Medicago sativa*.

#### References.

Ben-Dov et al. (2013), Moghaddam (2009) and Vahedi and Hodgson (2007).

### Porphyrophora tritici

(Bodenheimer)

Margarodes tritici Bodenheimer, 1941: 81.

#### Iran localities.

Ardabil, Hamadan, Kermanshah, Kordestan, Zanjan.

#### Host plants.

Poaceae: *Triticum aestivum*.

#### References.

Ben-Dov et al. (2013), Farahbakhsh (1961), Kozár et al. (1996), Moghaddam (2009), Moghaddam and Tavakoli (2010), Safar Alizadeh and Bahador (1987), Torabi et al. (2010), Vahedi (2001), Vahedi and Gholami Mahfar (2010) and Vahedi and Hodgson (2007).

### * Porphyrophora victoriae

Jashenko

Porphyrophora victoriae Jashenko, 1994: 36.

#### Iran localities.

Markazi.

#### Host plants.

Brassicaceae: *Cardaria draba*.

#### Note.

This the first record of *Porphyrophora victoriae* from Iran, identified by R. Jashenko.

## Family MONOPHLEBIDAE

### Drosicha turkestanica

Archangelskaya

Drosicha turkestanica Archangelskaya, 1931: 69.

#### Iran localities.

Khorasan -e Razvi.

#### Host plants.

Oleaceae: *Fraxinus* sp.

#### References.

Moghaddam (2009).

### Gueriniella serratulae

(Fabricius)

Coccus serratulae Fabricius, 1775: 744.

#### Iran localities.

Kermanshah, Lorestan.

#### Host plants.

Unknown plant.

#### References.

Moghaddam and Tavakoli (2009).

### Icerya purchasi

Maskell

Icerya purchasi Maskell, 1879: 221.

#### Iran localities.

Fars, Mazandaran, Tehran.

#### Host plants.

Aceraceae: *Acer* sp.; Fabaceae: *Glycine max*; Rutaceae: *Citrus* sp.; Thymelaeaceae: *Daphne odora*.

#### References.

Afchar (1937), Ben-Dov et al. (2013), Farahbakhsh (1961), Kaussari (1946, 1957), Kozár et al. (1996) and Moghaddam (2009).

### Pseudaspidoproctus gramineus

Jashenko & Danzig

Pseudaspidoproctus gramineus Jashenko & Danzig, 1992: 89.

#### Iran localities.

Unknown.

#### Host plants.

Poaceae.

#### References.

Kozár et al. (1996).

### Pseudaspidoproctus hyphaeniacus

(Hall)

Aspidoproctus hyphaeniacus Hall, 1925: 1.

#### Iran localities.

Sistan & Balouchestan.

#### Host plants.

Arecaceae: *Phoenix dactylifera*.

#### References.

Moghaddam (2009).

## Family ORTHEZIIDAE

### Orthezia urticae

(Linnaeus)

Aphis urticae Linnaeus, 1758: 453.

#### Iran localities.

Ardebil, Azarbaijan -e Garbi, Azarbaijan -e Sharghi, Chaharmahal-Bakhtiari, Esfahan, Fars, Golestan, Hamadan, Ilam, Kerman, Kermanshah, Khorasan -e Jonoubi, Khorasan -e Shomali, Kohgilouyeh & Boyerahmad, Kordestan, Lorestan, Sistan & Balouchestan, Zanjan.

#### Host plants.

Apiaceae: *Eryngium bungei*; Asteraceae: *Artemisia* sp., *Gundelia* sp., *Echinops ritro*, *Echinops persicae*; Fabaceae: *Astragalus* sp.; Thymelaeaceae: *Daphne angustifolia*.

#### References.

Ben-Dov et al. (2013), Bodenheimer (1944), Moghaddam (2009) and Moghaddam and Tavakoli (2010).

## Family PHOENICOCOCCIDAE

### Phoenicococcus marlatti

Cockerell

Phoenicococcus marlatti Cockerell, 1899: 262.

#### Iran localities.

Kerman, Kermanshah, Hormozgan, Sistan & Balouchestan, Yazd.

#### Host plants.

Arecaceae: *Phoenix dactylifera*.

#### References.

Afchar (1937), Ben-Dov et al. (2013), Farahbakhsh (1961), Kaussari (1946), Moghaddam (2004) and Seghatoleslami (1977).

## Family PSEUDOCOCCIDAE

### Adelosoma phragmitidis

Borchsenius

Adelosoma phragmitidis Borchsenius, 1948a: 584.

#### Iran localities.

Unknown.

#### Host plants.

Poaceae.

#### References.

Ben-Dov et al. (2013), Kozár et al. (1996) and Moghaddam (2013).

### Antonina crawi

Cockerell

Antonina crawi Cockerell, 1900: 70.

#### Iran localities.

Gilan.

#### Host plants.

Poaceae: *Bambusa* sp.

#### References.

Ben-Dov et al. (2013), Farahbakhsh (1961), Kozár et al. (1996) and Moghaddam (2013).

### Antonina graminis

(Maskell)

Sphaerococcus graminis Maskell, 1897: 244.

#### Iran localities.

Bushehr, Khouzestan.

#### Host plants.

Poaceae: *Poa* sp., *Saccharum officinarum*.

#### References.

Ben-Dov (1994), Ben-Dov et al. (2013), Hendricks and Kosztarab M. (1999), Kozár et al. (1996), Moghaddam (2006, 2009, 2013) and Williams (1970).

### Brevennia rehi

(Lindinger)

Ripersia rehi Lindinger, 1943: 152.

#### Iran localities.

Khouzestan.

#### Host plants.

Poaceae: *Echinochloa crus-galli*, *Oryza sativa*.

#### References.

Asadeh and Mosaddegh (1991), Ben-Dov et al. (2013), Kozár et al. (1996) and Moghaddam (2006, 2009, 2013).

### Ceroputo pilosellae

Šulc

Ceroputo pilosellae Šulc, 1898: 2. *Phenacoccus euphorbiaefolius* Bodenheimer, 1943.

#### Iran localities.

Azarbaijan -e Garbi, Hamadan, Ilam, Kermanshah.

#### Host plants.

Euphorbiaceae: *Euphorbia* sp.

#### References.

Ben-Dov et al. (2013), Bodenheimer (1944), Kaussari (1957), Kozár et al. (1996) and Moghaddam (2009, 2013).

### ● Chorizococcus viticola

Kaydan & Kozár

Chorizococcus viticola Kaydan & Kozár in Fallahzadeh et al. 2010: 158.

#### Iran localities.

Fars.

#### Host plants.

Vitaceae: *Vitis vinifera*.

#### References.

Ben-Dov et al. (2013), Fallahzadeh et al. (2010) and Moghaddam (2013).

### ● Coccidohystrix burumandi

Moghaddam

Coccidohystrix burumandi Moghaddam in Moghaddam and Alikhani 2009: 175.

#### Iran localities.

Markazi.

#### Host plants.

Euphorbiaceae: *Euphorbia* sp.

#### References.

Ben-Dov et al. (2013), Moghaddam and Alikhani (2009) and Moghaddam (2013).

### Dysmicoccus boninsis

(Kuwana)

Dactylopius (Pseudococcus) boninsis Kuwana, 1909: 161.

#### Iran localities.

Khouzestan.

#### Host plants.

Asteraceae: *Lactuca* sp.

#### References.

Asadeh and Mosaddegh (1991), Ben-Dov et al. (2013), Kozár et al. (1996) and Moghaddam (2006, 2009, 2013).

#### Note.

This is the first record of *Dysmicoccus boninsis* from the plant family Asteraceae.

### Dysmicoccus brevipes

(Cockerell)

Dactylopius brevipes Cockerell, 1893: 267.

#### Iran localities.

Sistan & Balouchestan.

#### Host plants.

Fabaceae: *Medicago sativa*.

#### References.

Ben-Dov et al. (2013) and Moghaddam (1999, 2004a, 2006, 2009, 2013).

### Eurycoccus tamariscus

Williams

Eurycoccus tamariscus Williams, 1984: 538.

#### Iran localities.

Sistan & Balouchestan.

#### Host plants.

Tamaricaceae: *Tamarix* sp.

#### References.

Ben-Dov et al. (2013), Moghaddam (2009, 2013) and Williams and Moghaddam (2007).

### ● Exallomochlus balouchestanensis

Moghaddam

Exallomochlus balouchestanensis Moghaddam, 2013: 25.

#### Iran localities.

Sistan & Balouchestan.

#### Host plants.

Anacardiaceae: *Mangifera indica*.

#### References.

Moghaddam (2013).

### Ferrisia virgata

(Cockerell)

Dactylopius virgatus Cockerell, 1893: 178.

#### Iran localities.

Sistan & Balouchestan.

#### Host plants.

Boraginaceae: *Cordia myxa*; Moraceae: *Ficus carica*, *Ficus religiosa*; Myrtaceae: *Myrtus communis*, *Psidium guajava*.

#### References.

Ben-Dov et al. (2013) and Moghaddam (2004a, 2006, 2009, 2013).

### Formicococcus robustus

(Ezzat & McConnell)

Planococcoides robustus Ezzat & McConnell, 1956: 59.

#### Iran localities.

Hormozgan.

#### Host plants.

Fabaceae: *Prosopis spicigera*.

#### References.

Ben-Dov et al. (2013) and Moghaddam (2004a, 2006, 2009, 2013).

### Kiritshenkella sacchari

(Green)

Ripersia sacchari Green, 1900a: 37.

#### Iran localities.

Khouzestan.

#### Host plants.

Poaceae: *Saccharum officinarum*.

#### References.

Ben-Dov et al. (2013), Kozár et al. (1996) and Moghaddam (2009, 2013).

### Maconellicoccus hirsutus

(Green)

Phenacoccus hirsutus Green, 1908: 25.

#### Iran localities.

Fars, Hormozgan, Sistan & Balouchestan.

#### Host plants.

Anacardiaceae: *Mangifera indica*; Arecaceae: *Phoenix dactylifera*; Combretaceae: *Terminalia catappa*; Convolvulaceae: *Ipomoea* sp.; Fabaceae: *Acacia arabica*, *Albizzia* sp., *Prosopis* sp., *Prosopis spicigera*; Lythraceae: *Lawsonia inermis*; Malvaceae: *Hibiscus rosa-sinensis*, *Hibiscus syriacus*; Moraceae: *Ficus riligiosa*, *Morus alba*; Myrtaceae: *Psidium guajava*, *Syzygium aromaticum*; Rhamnaceae: *Ziziphus spina-christi*; Rutaceae: *Citrus sinensis*; Salicaceae: *Salix* sp.; Tamaricaceae: *Tamarix* sp.

#### References.

Ben-Dov et al. (2013), Fallahzadeh et al. (2007) and Moghaddam (2006, 2009, 2013).

#### Note.

This is the first record of *Maconellicoccus hirsutus* from the plant family Tamaricaceae.

### Nipaecoccus filamentosus

(Cockerell)

Dactylopius filamentosus Cockerell, 1893a: 254.

#### Iran localities.

Ghazvin.

#### Host plants.

Moraceae: *Ficus* sp.

#### References.

Kaussari (1957) and Kozár et al. (1996).

### Nipaecoccus viridis

(Newstead)

Dactylopius viridis Newstead, 1894: 5.

#### Iran localities.

Bushehr, Fars, Hormozgan, Khouzestan, Kerman, Sistan & Balouchestan.

#### Host plants.

Acanthaceae: *Avicennia officinalis*; Apocynaceae: *Nerium oleander*; Fabaceae: *Prosopis spicigera*; Moraceae: *Morus alba*; Rhamnaceae: *Ziziphus spina-christi*; Rutaceae: *Citrus bigaradia*, *Citrus sinensis*; Tamaricaceae: *Tamarix* sp.; Ulmaceae: *Ulmus* sp.

#### References.

Asadeh and Mosaddegh (1991), Ben-Dov (1994), Ben-Dov et al. (2013), Kozár et al. (1996) and Moghaddam (2006, 2009, 2013).

### Paracoccus burnerae

(Brain)

Pseudococcus burnerae Brain, 1915: 111.

#### Iran localities.

Sistan & Balouchestan.

#### Host plants.

Acanthaceae: *Avecennia officinalis*.

References. Moghaddam (2013).

### Peliococcopsis priesneri

(Laing)

Phenacoccus priesneri Laing, 1936: 80.

#### Iran localities.

Fars.

#### Host plants.

Poaceae: *Cynodon dactylon*.

#### References.

Ben-Dov et al. (2013) and Moghaddam (2006, 2009, 2013).

### ● Peliococcus ilamicus

Moghaddam

Peliococcus ilamicus Moghaddam, 2013: 39.

#### Iran localities.

Ilam.

#### Host plants.

Fabaceae: *Prosopis stephaniana*.

#### References.

Moghaddam (2013).

### ● Peliococcus kimmericus

(Kiritshenko)

Phenacoccus kimmericus Kiritchenko, 1940: 189.

#### Iran localities.

Fars, Khouzestan.

#### Host plants.

Amaranthaceae: *Noaea* sp.; Fabaceae: *Prosopis stephaniana*.

#### References.

Ben-Dov et al. (2013), Kozár et al. (1996) and Moghaddam (2006, 2009, 2013).

#### Note.

This is the first record of *Peliococcus kimmericus* from the plant family Amaranthaceae.

### Peliococcus talhouki

Matile-Ferrero

Peliococcus talhouki Matile-Ferrero, 1984: 225.

#### Iran localities.

Khouzestan, Yazd.

#### Host plants.

Fabaceae: *Prosopis farcata*; Moraceae: *Morus alba*.

#### References.

Ben-Dov et al. (2013), Kozár et al. (1996) and Moghaddam (2009, 2013).

### Peliococcus turanicus

(Kiritshenko)

Phenacoccus turanicus Kiritshenko, 1932: 137.

#### Iran localities.

Tehran.

#### Host plants.

Brassicaceae: *Rhaphanus sativus*.

#### References.

Moghaddam (2002, 2009).

### Phenacoccus aceris

(Signoret)

Pseudococcus aesculi Signoret, 1875: 329.

#### Iran localities.

Kermanshah, Tehran.

#### Host plants.

Asteraceae: *Echinops ritro*; Moraceae: *Morus alba*; Rosaceae: *Crataegus azarollus*.

#### References.

Ben-Dov et al. (2013), Bodenheimer (1944), Farahbakhsh (1961), Kozár et al. (1996), Moghaddam (2009, 2010, 2013) and Moghaddam and Tavakoli (2010).

### Phenacoccus arthrophyti

Archangelskaya

Phenacoccus arthrophyti Archangelskaya, 1930: 78.

#### Iran localities.

Yazd.

#### Host plants.

Amaranthaceae: *Haloxylon* sp.

#### References.

Ben-Dov et al. (2013) and Moghaddam (2003, 2009, 2010, 2013).

### ● Phenacoccus betae

Moghaddam

Phenacoccus betae Moghaddam, 2010a: 65–67.

#### Iran localities.

Kermanshah, Markazi.

#### Host plants.

Amaranthaceae: *Amaranthus blitoides*, *Beta vulgaris*.

#### References.

Ben-Dov et al. (2013) and Moghaddam (2010a, 2013).

### Phenacoccus hordei

(Lindeman)

Westwoodia hordei Lindeman, 1886: 367.

#### Iran localities.

Tehran.

#### Host plants.

Poaceae.

#### References.

Moghaddam (2013).

### ● Phenacoccus karkasicus

Moghaddam

Phenacoccus karkasicus Moghaddam, 2013: 52.

#### Iran localities.

Esfahan.

#### Host plants.

Berberidaceae: *Berberis vulgaris*.

#### References.

Moghaddam (2013).

### ● Phenacoccus iranica

Moghaddam

Phenacoccus iranica Moghaddam, 2013: 54.

#### Iran localities.

Kerman.

#### Host plants.

Aceraceae: *Acer cinerascens*.

#### References.

Moghaddam (2013).

### Phenacoccus perillustris

Borchsenius

Phenacoccus perillustris Borchsenius, 1949: 215.

#### Iran localities.

Esfahan.

#### Host plants.

Berberidaceae: *Berberis vulgaris*.

#### References.

Ben-Dov et al. (2013) and Moghaddam (2010, 2013).

### Phenacoccus pumilus

Kiritchenko

Phenacoccus pumilus Kiritchenko, 1931: 314.

#### Iran localities.

Bushehr, Markazi, Sistan & Balouchestan, Yazd.

#### Host plants.

Amaranthaceae: *Halocharis sulpurea*; Asteraceae: *Centaurea virgata*; Brassicaceae: *Descuriana sophia*, *Lepidium latifolium*; Fabaceae: *Alhagi cameorum*; Geraniaceae: *Erodium neuradifolium*; Lamiaceae: *Salvia bracteata*.

#### References.

Moghaddam (2013).

### ● Phenacoccus salviacus

Moghaddam

Phenacoccus salviacus Moghaddam in Moghaddam and Alikhani 2010: 14.

#### Iran localities.

Markazi.

#### Host plants.

Lamiaceae: *Salvia bracteata*.

#### References.

Ben-Dov et al. (2013), Moghaddam (2010, 2013) and (Moghaddam and Alikhani (2010).

### ● Phenacoccus sherbinovskyi

Bodenheimer

Phenacoccus sherbinovskyi Bodenheimer, 1943: 32.

#### Iran localities.

Sistan & Balouchestan.

#### Host plants.

Lamiaceae: *Rydingia persica*.

#### References.

Ben-Dov et al. (2013), Bodenheimer (1944), Kaussari (1957), Kozár et al. (1996) and Moghaddam (2013).

### Phenacoccus solani

Ferris

Phenacoccus solani Ferris, 1918: 60.

#### Iran localities.

Esfahan, Fars.

#### Host plants.

Amaranthaceae: *Celosia cristata*; Asteraceae: *Chrysanthemum morifolium*; Poaceae: *Festuca arundinacea*.

#### References.

Ben-Dov et al. (2013), Moghaddam (2006, 2009, 2010, 2013), Moghaddam and Alikhani (2010) and Moghaddam et al. (2004).

### Phenacoccus solenopsis

Tinsley

Phenacoccus solenopsis Tinsley, 1898: 47.

#### Iran localities.

Bushehr, Hormozgan.

#### Host plants.

Malvaceae: *Hibiscus rosa-sinensis*.

#### References.

Ben-Dov et al. (2013), Moghaddam (2013) and Moghaddam and Bagheri (2011).

### Phenacoccus turanicus

Kiritchenko, 1932: 137.

#### Iran localities.

Esfahan, Markazi, Tehran.

#### Host plants.

Brassicaceae: *Descurainia sophia*, *Raphanus sativus*; Fabaceae: *Astragalus* sp.

#### References.

Ben-Dov et al. (2013) and Moghaddam (2009, 2013).

### Planococcus citri

(Risso)

Dorthesia citri Risso, 1813: 416. *Pseudococcus citri* Cockerell, 1902.

#### Iran localities.

Fars, Gilan, Golestan, Khouzestan, Markazi, Mazandaran, Tehran.

#### Host plants.

Apocynaceae: *Adenium obesum*, *Nerium oleander*; Arecaceae: *Chamacyparis lawsoniana*; Caryophyllaceae: *Dianthus barbatus*; Crassulaceae: *Crassula* sp.; Cupressaceae: *Cupressus* sp.; Ebenaceae: *Diospyros kaki*; Euphorbiaceae: *Codiaeum variegatum*, *Euphorbia pulcherrima*; Moraceae: *Ficus benjamina*, *Ficus carica*, *Ficus elastica*, *Morus alba*; Oleaceae: *Forsythia intermedia*, *Fraxinus excelsior*; Platanaceae: *Platanus orientalis*; Poaceae: *Oryza sativa*; Rutaceae: *Citrus bigaradia*, *Citrus sinensis*; Rosaceae: *Rosa* sp.; Strelitziaceae: *Strelitzia alba*.

#### References.

Afchar (1937), Asadeh and Mosaddegh (1991), Ben-Dov et al. (2013), Bodenheimer (1944), Farahbakhsh (1961), Kozár et al. (1996), Moghaddam (2006, 2009, 2010, 2013), William and Moghaddam (1999).

#### Note.

These are the first records of *Planococcus citri* from the plant families Caryophyllaceae, Crassulaceae and Cupressaceae.

### Planococcus ficus

(Signoret)

Dactylopius ficus Signoret, 1875: 315. *Pseudococcus vitis* Fernald, 1903.

#### Iran localities.

Elborz, Fars, Ghom, Khorasan -e Razavi, Markazi, Tehran.

#### Host plants.

Cucurbitaceae: *Citrullus vulgaris*; Lythraceae: *Punica granatum*; Moraceae: *Ficus carica*; Vitaceae: *Vitis persica*.

#### References.

Asadeh and Mosaddegh (1991), Ben-Dov (1994), Ben-Dov et al. (2013), Cox (1989), Kaussari (1946), Kozár et al. (1996), Moghaddam (2006, 2009, 2013) and Williams and Moghaddam (2000).

#### Note.

This is the first record of *Planococcus ficus* from the plant family Cucurbitaceae.

### Planococcus vovae

(Nasonov)

Pseudococcus (Dactylopius) vovae Nasonov, 1909: 484.

#### Iran localities.

Esfahan, Fars, Gilan, Golestan, Kerman, Tehran.

#### Host plants.

Cupressaceae: *Cupressus* sp.

#### References.

Ben-Dov (1994), Ben-Dov et al. (2013), Cox (1989), Cox and Ben-Dov (1986), Kozár et al. (1996), Lotfalizadeh and Ahmadi (2000), Moghaddam (2006, 2009, 2010, 2013) and Williams and Moghaddam (1999).

### ● Polystomophora arakensis

Moghaddam

Polystomophora arakensis Moghaddam in Moghaddam and Alikhani 2010: 12.

#### Iran localities.

Markazi.

#### Host plants.

Polygonaceae: *Atraphaxis* sp.

#### References.

Ben-Dov et al. (2013), Moghaddam (2010, 2013) and Moghaddam and Alikhani (2010).

### Pseudococcus comstocki

(Kuwana)

Dactylopius comstocki Kuwana, 1902: 52.

#### Iran localities.

Ghazvin, Gilan, Khorasan -e Shomali, Mazandaran, Tehran.

#### Host plants.

Fabaceae: *Robinia pseudoacacia*; Moraceae: *Morus alba*, *Morus nigra*.

#### References.

Ben-Dov et al. (2013), Kozár et al. (1996) and Moghaddam (2009, 2010, 2013).

### Pseudococcus cryptus

Hempel

Pseudococcus cryptus Hempel, 1918: 199.

#### Iran localities.

Unknown.

#### Host plants.

Unknown plant.

#### References.

Ben-Dov et al. (2013), Kozár et al. (1996) and Moghaddam (2013).

### Pseudococcus longispinus

(Targioni Tozzetti)

Dactylopius longispinus Targioni Tozzetti, 1867: 75. *Pseudococcus adonidum* Westwood, 1840.

#### Iran localities.

Gilan, Mazandaran, Tehran.

#### Host plants.

Buxaceae: *Buxus hyrcana*; Celastraceae: *Euonymus* sp.

#### References.

Ben-Dov (1994), Ben-Dov et al. (2013), Farahbakhsh (1961), Kaussari (1957), Kozár et al. (1996) and Moghaddam (2010, 2013).

#### Note.

This is the first record of *Pseudococcus longispinus* from the plant family Buxaceae.

### Pseudococcus viburni

(Signoret)

Dactylopius indicus Signoret, 1875: 317. *Pseudococcus affinis* Fernald, 1903. *Pseudococcus fathyi* Bodenheimer, 1944.

#### Iran localities.

Esfahan, Gilan, Tehran.

#### Host plants.

Amaranthaceae: *Amaranthus blitum*; Araceae: *Dieffenbachia* sp., *Phoenix* sp.; Bignoniaceae: *Catalpa speciosa*; Buxaceae: *Buxus hyrcana*; Cupressaceae: *Cupressus* sp.; Euphorbiaceae: *Codiaeum variegatum*; Fabaceae: *Albizzia* sp., *Cercis siliquastrum*; Ginkgoaceae: *Ginko biloba*; Lythraceae: *Punica granatum*; Moraceae: *Ficus carica*, *Morus alba*; Rosaceae: *Rosa* sp.; Solanaceae: *Solanum tuberosum*; Theaceae: *Camellia sinensis*; Vitaceae: *Vitis persica*.

#### References.

Ben-Dov et al. (2013), Ben-Dov (1994), Bodenheimer (1944), Kozár et al. (1996) and Moghaddam (2006, 2009, 2010, 2013).

#### Note.

These are the first records of *Pseudococcus viburni* from the plant families Amaranthaceae and Ginkgoaceae.

### ● Rhodania aeluropi

Williams & Moghaddam

Rhodania aeluropi Williams & Moghaddam, 2007: 38.

#### Iran localities.

Khouzestan.

#### Host plants.

Poaceae: *Aeluropus* sp.

#### References.

Ben-Dov et al. (2013), Moghaddam (2009, 2013) and Williams and Moghaddam (2007).

### Saccharicoccus sacchari

(Cockerell)

Dactylopius sacchari Cockerell, 1895: 195.

#### Iran localities.

Khouzestan.

#### Host plants.

Poaceae: *Saccharum officinalis*.

#### References.

Moghaddam (2013).

### Spilococcus alhagii

(Hall)

Pseudococcus alhagii Hall, 1926: 7.

#### Iran localities.

Esfahan, Fars, Khouzestan.

#### Host plants.

Lythraceae: *Punica granatum*; Rhamnaceae: *Ziziphus spina-christi*.

#### References.

Ben-Dov et al. (2013), Kozár et al. (1996), Moghaddam (2009, 2013) and Williams and Moghaddam (2007).

### Spilococcus flavus

(Borchsenius)

Pseudococcus flavus Borchsenius, 1949: 117.

#### Iran localities.

Unknown.

#### Host plants.

Poaceae.

#### References.

Ben-Dov et al. (2013), Kozár et al. (1996) and Moghaddam (2013).

### ● Spilococcus mirzayansi

(Moghaddam)

Chorizococcus mirzayansi Moghaddam, 2010: 64.

#### Iran localities.

Tehran.

#### Host plants.

Cactaceae: *Opuntia ficus-indica*.

#### References.

Ben-Dov et al. (2013), Moghaddam (2010a, 2013) and Tsai and Wu (2011).

### Trabutina crassispinosa

Borchsenius

Trabutina elastica Borchsenius, 1936: 111 [misidentification, according to Borchsenius 1941: 133]. *Trabutina crassispinosa* Borchsenius, 1941: 133.

#### Iran localities.

Ardabil, Kermanshah.

#### Host plants.

Tamaricaceae: *Tamarix* sp.

#### References.

Ben-Dov et al. (2013), Kozár et al. (1996), Moghaddam (2013) and Torabi et al. (2010).

### Trabutina mannipara

(Hemprich & Ehrenberg)

Coccus manniparus Hemprich & Ehrenberg in Ehrenberg 1829: 1.

#### Iran localities.

Esfahan, Khorasan -e Shomali.

#### Host plants.

Tamaricaceae: *Tamarix* sp.

#### References.

Ben-Dov et al. (2013), Danzig and Miller (1996) and Moghaddam (2013).

### Trabutina serpentina

(Green)

Naiacoccus serpentinus Green, 1919: 117. *Naiacoccus minor* Borchsenius, 1949. *Naiacoccus serpentinus* Green, 1919.

#### Iran localities.

Ardabil, Azarbaijan -e Garbi, Azarbaijan -e Sharghi, Bushehr, Hormozgan, Gilan, Kerman, Khouzestan, Sistan & Balouchestan, Zanjan.

#### Host plants.

Tamaricaceae: *Tamarix* sp.

#### References.

Ben-Dov et al. (2013), Bodenheimer (1944), Farahbakhsh (1961), Kozár et al. (1996) and Moghaddam (2006, 2009, 2010, 2013).

### Trionymus multivorus

(Kiritchenko)

Pseudococcus multivorus Kiritchenko, 1936: 151.

#### Iran localities.

Ardabil, Esfahan, Fars, Hamadan, Kerman, Kermanshah, Khorasan -e Shomali, Lorestan, Markazi.

#### Host plants.

Apiaceae: *Echinophora* sp.; Asteraceae: *Cirsium* sp., *Echinops ritro*, *Lactuca* sp., *Matricaria chamomilla*, *Klasea cerinthifolia*; Fabaceae: *Astragalus* sp.; Malvaceae: *Hibiscus* sp.

#### References.

Ben-Dov et al. (2013) and Moghaddam (2006, 2009, 2013).

### Vryburgia amaryllidis

(Bouché)

Coccus amaryllidis Bouché, 1837: 99. *Trionymus amaryllidis* Lindinger, 1934.

#### Iran localities.

Fars.

#### Host plants.

Amaryllidaceae: *Amaryllis* sp.

#### References.

Ben-Dov et al. (2013), Farahbakhsh (1961), Kozár et al. (1996) and Moghaddam (2013).

## Family PUTOIDEAE

### Puto superbus

(Leonardi)

Macrocerococcus superbus Leonardi, 1907: 152.

#### Iran localities.

Ardebil, Esfahan, Zanjan.

#### Host plants.

Fabaceae: *Astragalus* sp.

#### References.

Ben-Dov (1994), Ben-Dov et al. (2013), Borchsenius (1949), Danzig (1999), and Moghaddam (2009, 2013).

## Family RHIZOECIDAE

### Rhizoecus albidus

Goux

Rhizoecus (Pararhizoecus) albidus Goux, 1942: 40.

#### Iran localities.

Esfahan, Markazi, Tehran.

#### Host plants.

Cactaceae: *Gymnocalycium baldianium*; Crassulaceae: *Echeveria* sp.; Poaceae: *Festuca arundinacea*.

#### References.

Ben-Dov et al. (2013) and Moghaddam (2001, 2009, 2013).

#### Note.

These are the first records of *Rhizoecus albidus* from the plant families Cactaceae and Crassulaceae.
